# Phosphoproteomic analysis of anaplastic lymphoma kinase (ALK) downstream signaling pathways identifies signal transducer and activator of transcription 3 as a functional target of activated ALK in neuroblastoma cells

**DOI:** 10.1111/febs.12453

**Published:** 2013-08-22

**Authors:** Kamaraj Sattu, Falko Hochgräfe, Jianmin Wu, Ganesh Umapathy, Christina Schönherr, Kristina Ruuth, Damini Chand, Barbara Witek, James Fuchs, Pui-Kai Li, Fredrik Hugosson, Roger J Daly, Ruth H Palmer, Bengt Hallberg

**Affiliations:** 1 Department of Molecular Biology, Umeå UniversitySweden; 2 Competence Center Functional Genomics, University of GreifswaldGermany; 3 Cancer Research Program, Garvan Institute of Medical ResearchSydney, New South Wales, Australia; 4 St Vincent's Clinical School, Faculty of Medicine, University of New South WalesNew South Wales, Australia; 5 Division of Medicinal Chemistry and Pharmacology, College of Pharmacy, Ohio State UniversityColumbus, OH, USA; 6 Signalling Network Laboratory, Department of Biochemistry and Molecular Biology, School of Biomedical Sciences, Monash UniversityVictoria, Australia

**Keywords:** anaplastic lymphoma kinase, cancer, neuroblastoma, SHP-2, signal transducer and activator of transcription 3 (STAT3)

## Abstract

Activation of the anaplastic lymphoma kinase (ALK) receptor tyrosine kinase is a key oncogenic mechanism in a growing number of tumor types. In the majority of cases, ALK is activated by fusion with a dimerizing partner protein as a result of chromosomal translocation events, most studied in the case of the nucleophosmin–ALK and echinoderm microtubule-associated protein-like 4–ALK oncoproteins. It is now also appreciated that the full-length ALK receptor can be activated by point mutations and by deletions within the extracellular domain, such as those observed in neuroblastoma. Several studies have employed phosphoproteomics approaches to find substrates of ALK fusion proteins. In this study, we used MS-based phosphotyrosine profiling to characterize phosphotyrosine signaling events associated with the full-length ALK receptor. A number of previously identified and novel targets were identified. One of these, signal transducer and activator of transcription 3 (STAT3), has previously been observed to be activated in response to oncogenic ALK signaling, but the significance of this in signaling from the full-length ALK receptor has not been explored further. We show here that activated ALK robustly activates STAT3 on Tyr705 in a number of independent neuroblastoma cell lines. Furthermore, knockdown of STAT3 by RNA interference resulted in a reduction in myelocytomatosis neuroblastom (MYCN) protein levels downstream of ALK signaling. These observations, together with a decreased level of MYCN and inhibition of neuroblastoma cell growth in the presence of STAT3 inhibitors, suggest that activation of STAT3 is important for ALK signaling activity in neuroblastoma.

## Introduction

Many of the tyrosine kinases in the human kinome are implicated in human cancers 1, and provide important targets for cancer treatment, as well as biomarkers for patient stratification. Recently, tyrosine kinase inhibitors targeting anaplastic lymphoma kinase (ALK) have been approved for the treatment of ALK-positive non-small-cell-lung-cancer (NSCLC) 2. In NSCLC, ALK activation occurs via chromosomal translocation, leading to activation of the kinase domain. However, ALK is also known to be mutated in the context of the full-length receptor tyrosine kinase (RTK), this being most clearly understood in neuroblastoma. Neuroblastoma is a childhood cancer that stems from the sympathetic nervous system 3, most commonly originating in the adrenal glands, but also developing at additional sites in the neck, chest, and abdomen. It is considered to be a disease of developing tissue, as it originates from precursor cells of neural crest tissue that are active during embryonic development 4. ALK gain-of-function mutations have been described in both familial 5–6 and sporadic 6–10 neuroblastoma. Although there is currently no clinically approved specific treatment for ALK mutations in neuroblastoma, ongoing clinical trials are being conducted to determine the potential usefulness of ALK-targeted therapies for use in future treatment approaches 11. Today, crizotinib (PF-02341066) is in use as a Food and Drug Administration-approved drug for the treatment of ALK-positive NSCLC 12. Clinical studies today suggest that anti-ALK inhibitor therapy offers promise in the treatment of ALK-mediated tumors 2,11.

One consistent problem with kinase inhibitors and their use in a more personalized therapy approach is the drug-resistant mutations that arise in response to treatment, most commonly within the ATP-binding site of the kinase in question. Originally described in the epidermal growth factor receptor and breakpoint cluster region–c-abl kinase domains 14–18, this has now also been described for echinoderm microtubule-associated protein-like 4 (EML4)–ALK by Choi *et al*., and others who have described the appearance of such mutations in the fusion EML4–ALK protein that confers resistance to crizotinib 19–22. Phase I/II studies of crizotinib in children with relapsed/refractory tumors involving ALK, including neuroblastoma patients, have been initiated (ClinicalTrials.gov, NCT01182896) 23.

Given the increasing clinical importance of ALK activity, we chose to characterize ALK-mediated tyrosine kinase signaling networks by MS-based phosphoproteomics. In the current study, we conducted a comprehensive profiling of endogenous proteins that are tyrosine-phosphorylated upon expression and activation of wild-type ALK in PC12 cells, a cell line originally from the rat neural crest. This list, which reflects both direct and indirect tyrosine phosphorylation targets as a result of ALK activity within the cell, verifies several recently published results such as fibroblast growth factor receptor substrate 2 (FRS2) 23. One of the proteins that was most significantly tyrosine-phosphorylated in response to ALK activation in our analysis was signal transducer and activator of transcription 3 (STAT3), which has previously been reported to be phosphorylated by both the full-length ALK and ALK fusion proteins 24–27. Here, we investigated this further, and clarified the importance of STAT3 as a mediator in the initiation of transcription of *MYCN*.

## Results

### Phosphotyrosine profiling of PC12 cells expressing ALK

**Figure 1:**
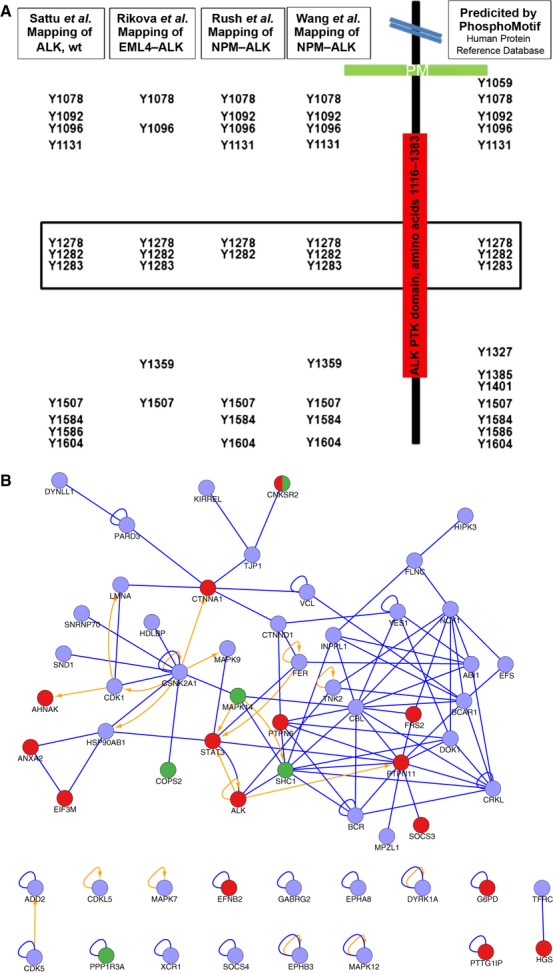
(A) Tyrosine residues phosphorylated in the kinase domain of ALK. The intracellular domain of ALK containing the protein kinase domain (PKD) (red) and potential autophosphorylation sites were searched with phosphomotif (http://www.hprd.org/PhosphoMotif_finder) as indicated. Presented and compared side-by-side with our phosphotyrosine mapping of activated full-length ALK are the global surveys of phosphotyrosine peptides identified in EML4–ALK and NPM–ALK 29,30. The critical tyrosines in the activation loop of the kinase domain of ALK are boxed 45. (B) Protein–protein interactions of human orthologs of the phosphoproteins identified in ALK-expressing PC12 cells. In the network, proteins with upregulated phosphorylation sites in activated ALK-expressing PC12 cells as compared with control cells are in red, and proteins with downregulated phosphorylation sites are in green. Blue edges indicate protein–protein interactions, and orange edges indicate kinase–substrate relationships. Only the network including ALK is shown. The pale blue balls indicate (human orthologs of) PC12 proteins with identified/mapped phosphotyrosines that were not found to be significantly regulated.

To identify sites with altered phosphorylation associated with ALK RTK activity, we utilized an immunoaffinity-coupled LC-MS/MS approach on PC12 Tet-on-inducible cell lines. After induction, ALK was activated by addition of agonist mAbs that have previously been reported to initiate ALK signaling pathways 10–28. Comparison with controls expressing unstimulated ALK allowed quantification of 336 phosphorylation sites (207 phosphotyrosine, 78 phosphoserine, and 51 phosphothreonine) derived from 189 different proteins (Table S1). Label-free quantification of ALK-activated versus control cells revealed 101 phosphorylation sites (73 phosphotyrosine, 13 phosphoserine, and 15 phosphothreonine) in 72 proteins, with a more than five-fold increase in corresponding phosphopeptide intensities. In contrast, only 19 phosphorylation sites in 12 proteins were found with decreased phosphorylation following ALK induction. Within the list of mapped phosphorylation sites, we identified 11 phosphotyrosine sites that belong to ALK (Fig. 1A). As expected, all of them showed a significant increase in the level of phosphorylation on activation of ALK with mAb as compared with control cells (Table S1). The identified tyrosine phosphorylation sites in full-length ALK overlap with the predicted ones (PhosphoMotif, Human Protein Reference Database, http://www.hprd.org/PhosphoMotif_finder), and cover, with only one exception, the entire set of mapped phosphotyrosine sites from earlier investigations of EML4–ALK and nucleophosmin (NPM)–ALK 29,30.

To highlight potential direct downstream substrates of full-length ALK, a network analysis using known protein–protein interactions and experimentally verified kinase–substrate relationships of the human orthologs of the identified phosphoproteins was performed (Fig. 1B). The network model illustrated two of the identified phosphoproteins with increased phosphorylation as potential direct targets of ALK following ALK induction, namely PTPN11 (SHP-2) and STAT3. Neither of these has previously been shown to interact with full-length ALK. In agreement with a role for these proteins downstream of ALK, we detected approximately 10-fold higher phosphorylation at Tyr546 and Tyr584 in SHP-2 as well as Tyr705 in STAT3 under ALK-activating conditions in our model. Furthermore, identified downstream targets of ALK detected in this phosphoprofiling, such as mitogen-activated protein kinase (MAPK)1, MAPK3 [extracellular signal-related kinase (ERK)2/1], glycogen synthase kinase-3α, STAT3, FAK, and CRKL (Table S1), showed decreased phosphorylation upon abrogation of ALK activity in several neuroblastoma cell lines (Fig. S1).

### ALK activates STAT3 in PC12 cells

**Figure 2:**
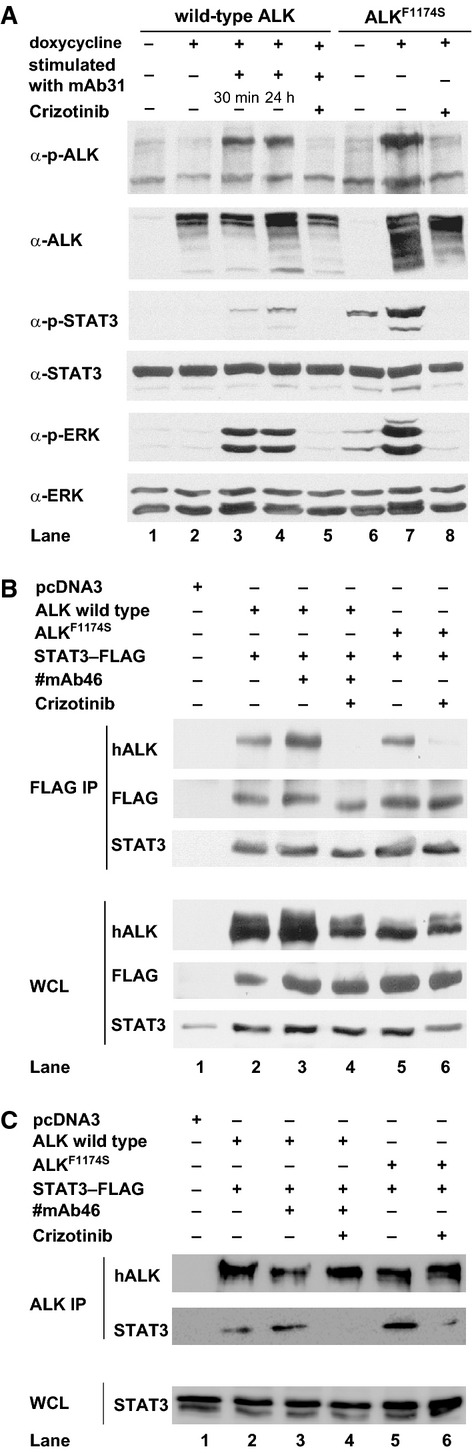
STAT3 phosphorylation and interaction with ALK on ALK activation. (A) Tet-on-inducible PC12 cell clones expressing either wild-type ALK or the ALK^F1174S^ mutant receptor were employed. Protein expression was induced with 1 μg·mL^−1^ doxycycline, and cells were serum-starved for 24 h prior to stimulation with 1 μg·mL^−1^ ALK-activating mAb (mAb31) for 30 min or 24 h. Whole cell lysates were analyzed by SDS/PAGE, and this was followed by immunoblotting with antibodies against p-ALK^Y1278^, ALK, p-STAT3^Y705^, and p-ERK. Pan-ERK and pan-STAT3 antibodies were employed as loading controls. (B, C) PC12 cells were transfected with either wild-type ALK or the ALK^F1174S^ mutant together with FLAG-tagged STAT3 prior to stimulation with 1 μg·mL^−1^ ALK-activating mAb (mAb46) for 24 h, in the presence or absence of 250 nm crizotinib, as indicated. Lysates were immunoprecipitated (IP) with either antibody against FLAG (M2) (B) or with antibody against ALK (mAb31) (C), and this was followed by immunoblotting for ALK, FLAG, and STAT3, as indicated. WCL, whole cell lysate.

We subsequently decided to focus in more detail on one of the most prominent hits from the phosphotyrosine proteomics screen, namely STAT3. As observed in the screen, activation of ALK resulted in clear phosphorylation of STAT3 at Tyr705. PC12 cells expressing either doxycycline-inducible wild-type ALK or the ALK^F1174S^ mutant were employed to examine STAT3 activation. As ALK is still regarded as an orphan receptor, the ALK receptor was stimulated with an agonist mAb that has been previously reported to bind and activate ALK in a cell culture model 28,32. Interestingly, whereas long-term stimulation of the wild-type ALK receptor did lead to visible tyrosine phosphorylation of STAT3, this was not prominently observed after 30 min of stimulation, a time point when ALK and ERK1/2 were highly phosphorylated (Fig. 2A, compare lanes 3 and 4). This is in contrast to the robust activation of STAT3 observed upon induction of expression of the ALK^F1174S^ mutant (Fig. 2A, compare lanes 3 and 7). In both cases, tyrosine phosphorylation of STAT3 was abrogated by the addition of the ALK inhibitor crizotinib (Fig. 2A, compare lanes 4 and 7 with lanes 5 and 8).

To investigate the activation process further, we examined whether an interaction between ALK and STAT3 could occur. We were unable to observe an interaction between doxycycline-induced ALK and endogenous STAT3 in PC12 cells (data not shown). However, an interaction between STAT3 and ALK was observed on immmunoprecipitation of tagged STAT3, when wild-type ALK was transiently cotransfected with FLAG-tagged STAT3 (Fig. 2B). Upon stimulation of ALK, increased interaction between ALK and STAT3 was observed (Fig. 2B, lane 3). This interaction was abrogated by addition of the ALK inhibitor crizotinib (Fig. 2B, lane 4). Similarly, on cotransfection of activated ALK^F1174S^ with FLAG-tagged STAT3, an interaction was observed that was blocked upon addition of crizotinib prior to immunoprecipitation (Fig. 2B, compare lanes 5 and 6). Similarly, we were able to observe STAT3 in both wild-type and gain-of-function ALK immunoprecipitates from PC12 cells transiently expressing ALK and STAT3 (Fig. 2C). In agreement with our previous observations, the STAT3–ALK interaction was crizotinib-sensitive (Fig. 2C, compare lanes 3 and 5 with lanes 4 and 6). Quantification of independent blots showed clear six-fold to 10-fold increased binding of STAT3 to ALK upon stimulation or when a gain-of-function ALK variant was employed (Fig. S2). These data suggest that, upon activation, STAT3 may be recruited to ALK signaling complexes. However, it is important to note that our attempts to verify this interaction at the level of endogenous proteins in neuroblastoma cell lines have not been successful.

### STAT3 is important for MYCN expression in response to ALK activation

**Figure 3:**
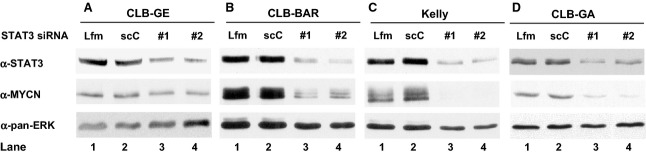
Loss of STAT3 results in reduced MYCN levels. Two independent STAT3 siRNAs (#1 or #2) were employed to downregulate STAT3 levels in CLB-GE (A), CLB-BAR (B), Kelly (C) and CLB-GA (D) neuroblastoma cell lines. Cells were transfected with either control scrambled siRNA, STAT3 siRNA#1 or STAT3 siRNA#2 prior to cell lysis 48 h post-transfection. Whole cell lysates were subsequently immunoblotted for STAT3, MYCN, and pan-ERK (as loading control), as indicated. Lfm, lipofectamine; scC, scramble control.

Given recent observations that ALK regulates *MYCN* transcription in neuroblastoma cells and collaborates with MYCN in neuroblastoma pathogenesis 34–37, we decided to investigate a role for STAT3 in this process. Initially, we employed small interfering RNA (siRNA) targeting STAT3 in a number of neuroblastoma cell lines, including CBL-GE, CBL-BAR, CBL-GA and Kelly cells. These neuroblastoma cell lines are all ALK gain-of-function in nature, containing either an activated ALK mutation (ALK^R1275Q^, CBL-GA; ALK^F1174V^, CLB-GE; ALK^F1174L^, Kelly) or overexpressing an ALK receptor with an extracellular domain deletion (CLB-BAR^Δexon 4–12^), and express different levels of MYCN (Fig. S3) 38,39. Cell lines were transfected with either scrambled siRNA, two independent siRNAs targeting STAT3, or a mock control. In each cell line tested, the scrambled siRNA transfection did not reduce STAT3 levels, which were comparable to those in cells with control transfection without siRNA. However, upon transfection with specific STAT3 siRNA, all cell lines tested showed reduced levels of STAT3 as compared with the scrambled transfection controls (Fig. 3A–D, top panels, compare lanes 3 and 4 with lane 2). Furthermore, a clear reduction in MYCN levels in CLB-BAR, CLB-GA and Kelly cells was observed upon treatment with siRNA targeting STAT3 (Fig. 3A–D, middle panels, compare lanes 3 and 4 with lane 2).

**Figure 4:**
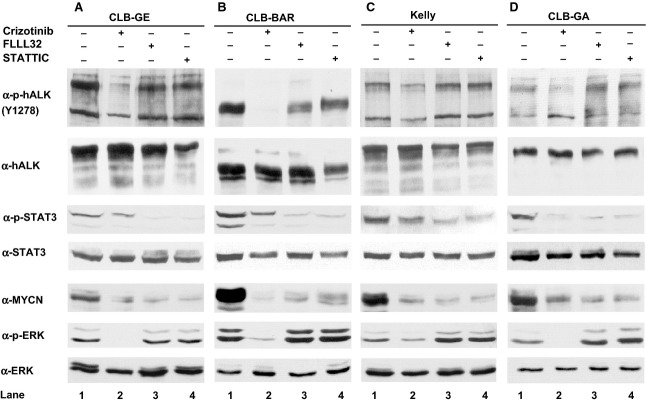
STAT3 activity is required for regulation of MYCN expression by ALK. Neuroblastoma cell lines CLB-GE (A), CLB-BAR (B), Kelly (C) and CLB-GA (D) were starved and treated with either 250 nm crizotinib (24 h), 5 μm FLLL32 (8 h), 5 μm STATTIC (8 h), or control, as indicated. After cell lysis, samples were immunoblotted with antibodies against p-ALK^Y1278^, MYCN, p-STAT3^Y705^, and p-ERK. Pan-ERK, ALK and STAT3 antibodies were employed as loading controls. Three independent experiments with similar results were performed, and representative blots are shown.

To further validate these results, we employed a number of STAT3 inhibitors, including FLLL32 and STATTIC, which have previously been shown to inhibit STAT3 activation 41,42. We investigated ALK, STAT3 and MYCN levels in CLB-GE, CLB-BAR, Kelly and CLB-GA neuroblastoma cell lines upon treatment with STAT3 inhibitors (Fig. 4A–D). Treatment with either FLLL32 or STATTIC efficiently abrogated the phosphorylation of STAT3 without affecting general STAT3 levels. Importantly, whereas these inhibitors blocked STAT3 activity, they did not affect the phosphorylation status of ERK or ALK itself (Fig. 4A–D, compare lanes 3 and 4 with lane 1). In keeping with the results obtained with STAT3 siRNA treatment, both inhibitors reduced MYCN levels (Fig. 4A–D, compare lanes 3 and 4 with lane 1), suggesting that STAT3 may act between the ALK receptor and expression of MYCN. The ALK inhibitor crizotinib was employed as a control, leading to a reduction in the phosphorylation of STAT3 and ALK, expression of MYCN and phosphorylation of ERK (Fig. 4A–D, compare lane 2 with lanes 1, 3 and 4).

**Figure 5:**
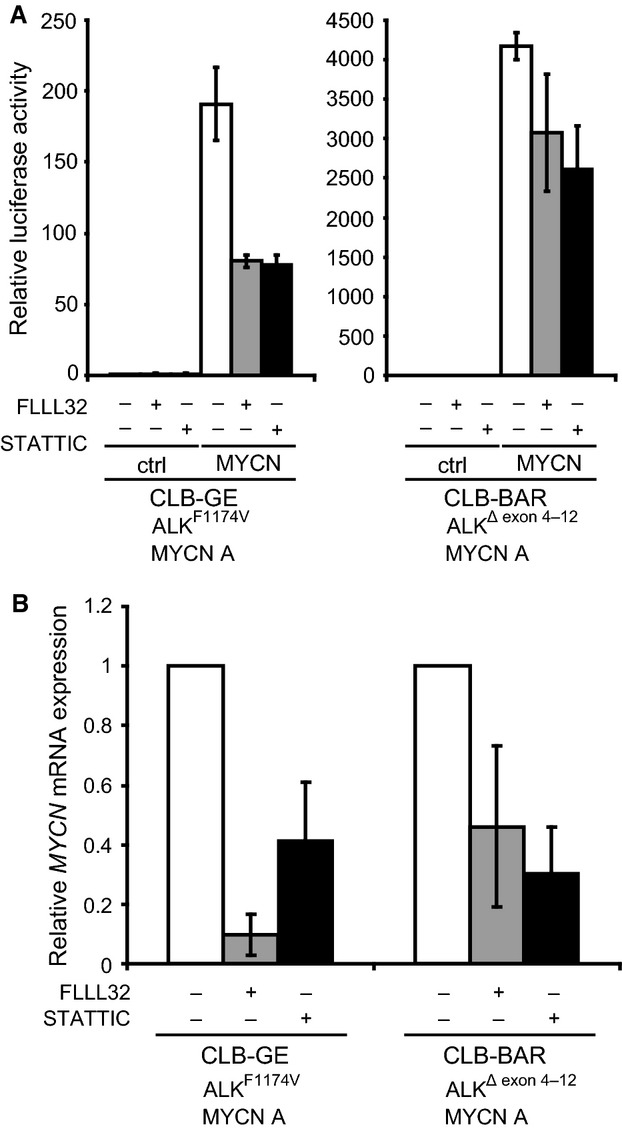
Inhibition of STAT3 reduces *MYCN* transcription. (A) Luciferase assay of neuroblastoma cell lines transfected with *MYCNP*–luciferase or empty pGL2 vector as a control (ctrl). The neuroblastoma cell lines CLB-GE and CLB-BAR were transfected with empty pGL2 (ctrl) or *MYCNP*–luciferase. Cells were then serum-starved, and STAT3 was inhibited with 2.5 μm FLLL32 or STATTIC for 12 h. White bars: untreated neuroblastoma cells. Gray bars: cells treated with FLLL32. Black bars: cells treated with STATTIC. Results are presented as relative luciferase activity, where untreated samples transfected with empty pGL2 vector were set to 1. (B) qRT-PCR of *MYCN* mRNA in neuroblastoma cell lines. The neuroblastoma cell lines CLB-GE and CLB-BAR were starved and treated with 2.5 μm FLLL32 or STATTIC for 12 h. Primers amplifying part of the coding sequence of RPL19 (B) or RPL29 (data not shown) were used to control for differences in cDNA input. Relative expression was calculated according to the ΔΔCt relative quantification method. Each sample within an experiment was analyzed in duplicate, and the experiment was carried out at least three times. White bars: untreated cells. Gray bars: cells treated with FLLL32. Black bars: cells treated with STATTIC.

To investigate further whether STAT3 is involved in ALK-activated initiation of *MYCN* transcription, we employed an MYCNP–luciferase assay in two independent neuroblastoma cell lines 36. Cells were transfected with *MYCNP*–luciferase reporter or control, and treated with either STATTIC or FLLL32. Upon treatment with STAT3 inhibitors, both cell lines showed reduced luciferase activity as compared with untreated cells (Fig. 5A). Additionally, we employed quantitative RT-PCR (qRT-PCR) to confirm a role for STAT3 in ALK regulation of *MYCN* transcription. As controls, we employed primers amplifying part of the coding sequence of ribosomal protein 29 (RPL29) or ribosomal protein 19 (RPL19) (Fig. 5B, data not shown). Neuroblastoma cell lines treated with STATTIC or FLLL32 for 24 h showed a significant reduction in *MYCN* mRNA levels in comparison with untreated cells (Fig. 5B). Thus, pharmacological inhibition of STAT3 activity in neuroblastoma cell lines harboring ALK gain-of-function mutations results in reduced transcription of *MYCN* mRNA.

### STAT3 activity is required for growth and viability of neuroblastoma cells

**Figure 6:**
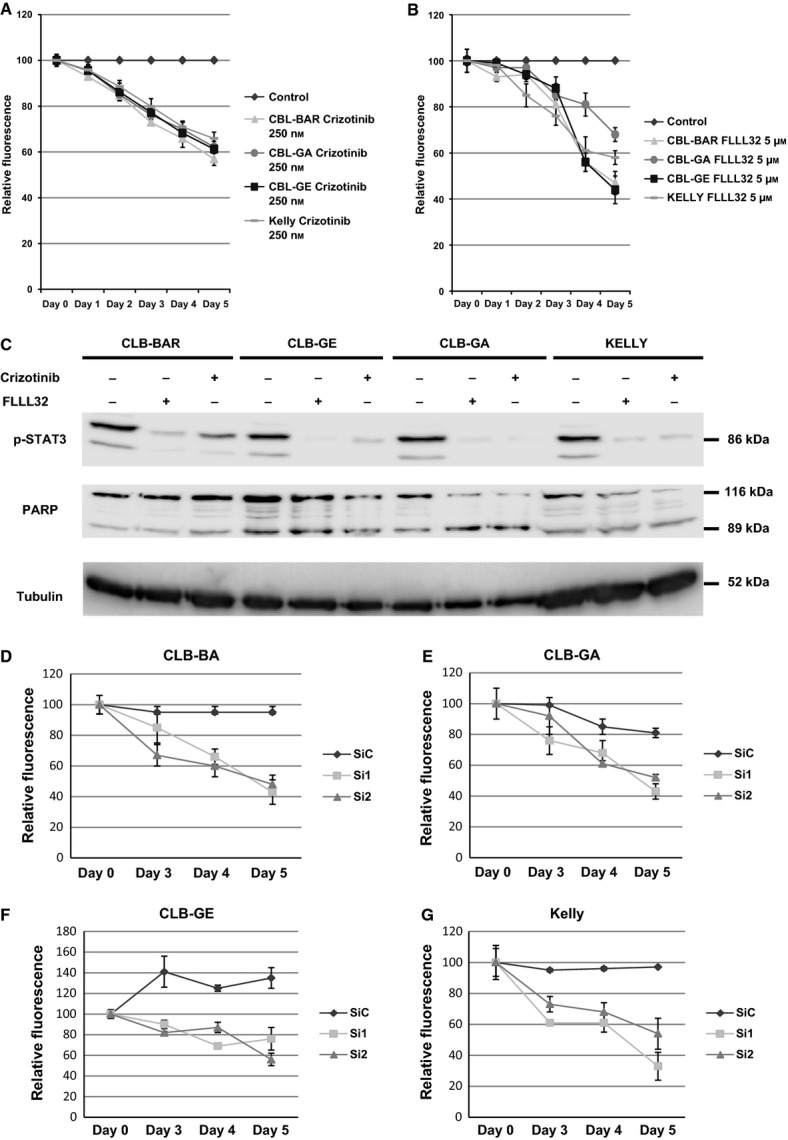
Loss of STAT3 function decreases neuroblastoma cell proliferation. (A, B) Neuroblastoma cell lines CLB-GE (▪), CLB-BAR (▴), Kelly (‐) and CLB-GA (•) were treated with 250 nm crizotinib (A) and 1.5 μm FLLL32 (B) for 5 days. Proliferation was analyzed with the resazurin cell proliferation assay. Values are reported as fold relative fluorescence from FLLL32-treated cells versus relative fluorescence from untreated cells (♦). Results are from three representative experiments, with each experiment performed in triplicate. (C) Neuroblastoma cell lines CLB-BAR, CLB-GE, CLB-GA and Kelly were grown on six-well plates with complete growth medium, starved, and treated with 250 nm crizotinib and 1.5 μm FLLL32 for 6 h. Cell lysates were immunoblotted with antibodies against p-STAT3 and PARP. Tubulin was used as a loading control. (D–G) CLB-BAR (D), CLB-GA (E) CLB-GE (F) and Kelly (G) cell lines were transfected with scrambled siRNA (SiC) (▪), STAT3 siRNA#1 (Si1) (▴) or STAT3 siRNA#2 (Si2) (▴) at 0 and 24 h. Cell viability was assessed at 0, 3, 4 and 5 days post-transfection, with the resazurin assay. Values are reported as fold relative fluorescence from siRNA-transfected cells versus relative fluorescence from control mock-transfected cells. Results are from one of three representative experiments, with each experiment performed in triplicate.

We then investigated whether STAT3 is not only important for initiation of *MYCN* transcription but also might influence the proliferation of our neuroblastoma cell lines. In this analysis, we employed CLB-GE, CLB-BAR, Kelly and CLB-GA neuroblastoma cell lines, measuring their growth in response to treatment with the STAT3 inhibitor FLLL32 or STAT3 siRNA. In all cell lines examined, reduction of endogenous STAT3 expression levels by siRNA or treatment with the STAT3 inhibitor FLLL32 resulted in a decrease in cell growth as compared with untreated cells (Fig. 6B, D–G) in a similar manner as crizotinib decreased cell growth (Fig. 6A). Furthermore, it was clear that treatment of neuroblastoma cell lines with either FLLL32 or crizotinib reduced the phosphorylation status of STAT3 to a similar degree, without increasing cleavage of poly(ADP-ribose) polymerase (PARP), which was used as a measure of apoptosis (Fig. 6C).

## Discussion

It is interesting to compare the results of this study with earlier phosphoproteomics analysis on oncogenic ALK fusion proteins, such as EML4–ALK and NPM–ALK 29,30. In terms of phosphorylation of ALK itself, these studies identified overlapping but not identical sites in the ALK intracellular domain. Activation of full-length ALK results in phosphorylation of Tyr1278, Tyr1282, and Tyr1283, which lie in the Y′RAS′YY autophosphorylation motif in the activation loop. Although our data do not allow us to comment on the order of phosphorylation within the Y′RAS′YY motif, they are in agreement with previous studies elucidating the mechanism of phosphorylation 44,45, and further support the use of antibodies against pTyr1278 as an indicator of ALK activation. Another site commonly used to denote activation of the ALK receptor is Tyr1604, which is situated at the C-terminal tail of the receptor, and has been reported to be important for transformation activity and docking of phospholipase Cγ in studies on NPM–ALK 47. Although antibodies against pTyr1604 are commonly used to assay ALK activity, the importance of Tyr1604 in the interaction of phospholipase Cγ with full-length ALK has not been confirmed. The identification in this study reinforces the potential importance of this tyrosine in ALK activation. Of the phosphorylated tyrosines identified outside of the activation loop, Tyr1507 of ALK lies within a consensus Shc-binding site (NPTpY), and has been shown to be critical for interaction of full-length ALK with Shc 23. These results are consistent with earlier work on NPM–ALK 48. The remaining tyrosines that are phosphorylated in response to ALK activation identified in this study, namely Tyr1078, Tyr1092, Tyr1096, Tyr1131, Tyr1584, and Tyr1586, have been poorly characterized in the context of the full-length ALK RTK, and determination of their significance requires further analysis. However, work on the oncogenic ALK fusion proteins implicates these residues in binding of IRS-1 (Tyr1096) 48, and SNT (FRS2) (Tyr1096 and Tyr1507) 49. As well as identifying tyrosine phosphorylation sites in ALK, we observed phosphorylation of Ser1086, Ser1281 and Thr1597 on ALK. Although this finding is intriguing, the significance of these phosphorylation events is unclear. Although the phosphorylation of RTKs on serine and threonine is well recognized, its role is much less understood than that of tyrosine phosphorylation. However, serine/threonine phosphorylation is known to have important regulatory functions for, for example, fibroblast growth factor receptor, epidermal growth factor receptor 50–51, and Kit 52, to name but a few.

Although information on the phosphorylation of ALK itself in response to activation represented an important part of the information obtained with this phosphoproteomics approach, the identification of candidate molecules phosphorylated in response to ALK activation was the primary aim of this investigation. The list of phosphoproteins contains both known and novel ALK signaling components, some of which have been characterized, but the majority of which remain to be investigated for their relevance in both physiological and pathological ALK signaling. Of those known candidates, several examples can be briefly mentioned. One molecule identified in our screen was FRS2, which was phosphorylated on Tyr349 in response to ALK activation. FRS2 has previously been reported to bind full-length ALK, although the binding site is unclear 23. Similarly, MAPK1 – which was phosphorylated on Thr183 in this study – has been shown by several groups to be phosphorylated upon stimulation of the ALK receptor 53.

One of the major tyrosine-phosphorylated proteins identified in this study was STAT3, which was phosphorylated on Tyr705 in response to ALK activation. STAT3 has been reported to be phosphorylated downstream of several of the oncogenic ALK fusion proteins 25,27. To date, although several articles have reported STAT3 phosphorylation downstream of full-length ALK, the significance of this is unclear 8–56. One study of NPM–ALK has suggested that oncogenic ALK binds and activates STAT3 directly via the first tyrosine (Tyr1278) in the Y′RAS′YY motif of ALK 45. The crystal structures of ALK show that unphosphorylated Tyr1278 interacts with Cys1097 in the N-lobe of the kinase, implying that phosphorylation of Tyr1278 should make a binding site for STAT3 available, consistent with this hypothesis. In this study, we saw clear phosphorylation of STAT3 on Tyr705, which is particularly robust in response to oncogenic forms of the full-length ALK receptor, such as ALK^F1174S^, ALK^Δexon 2–3^, and ALK^Δexon 4–12^ 39,55. Although it was not investigated in this study, it would be of interest to examine the importance of STAT3 phosphorylation in neuroblastoma cells harboring a wild-type nonactive ALK receptor. This hypothesis is supported by inhibition of the NB1 neuroblastoma cells lines with crizotinib, which resulted in a loss of pSTAT3^Y705^, although the significance of STAT3 for cell growth was not discussed 54. In light of the activation of STAT3 by ALK, we also investigated the interaction of ALK with STAT3. Indeed, we observed an interaction of STAT3 with full-length ALK when overexpressed; however, this was not detectable at the level of endogenous proteins, suggesting that care should be taken when interpreting these results. It is unclear whether this is because of limitations of the antibodies employed here, or a masking of the epitope by the interaction, or simply a low level of endogenous interaction. Although the precise molecular mechanisms underlying ALK activation of STAT3 are unknown, it is clear from these studies that loss of STAT3 activity, either by RNA interference-mediated knockdown of STAT3 or by addition of STAT3 inhibitors 41,42, reduces the growth and viability of four neuroblastoma lines. One key component examined here was the effect of STAT3 activity on MYCN levels. Recent work has highlighted the cooperative roles of ALK and MYCN in neuroblastoma, with ALK impacting on both the level of *MYCN* transcription and the stability of the MYCN protein itself 34–37. Given these reports, our finding here that STAT3 is important for changes in MYCN levels in response to ALK signaling is significant. Indeed, inhibition of STAT3 by addition of inhibitors, or by RNA interference, reduced MYCN levels even in the presence of activated ALK.

In conclusion, our phosphoproteomic analysis identifies a number of phosphorylation sites targeted by activation of the ALK RTK. One prominent target identified in this analysis was STAT3, which we show here is important for the regulation of MYCN downstream of activated ALK. From our investigations, it is also clear that there is a difference between the ability of wild-type ALK to activate STAT3 and that of oncogenically activated forms of ALK. The molecular mechanisms underlying this difference are not known, and further work is required to understand this. This work suggests that STAT3 inhibition may be a viable approach in the regulation of MYCN activity in neuroblastoma cells, and may have potential therapeutic value in the future.

## Experimental procedures

### Phosphotyrosine profiling

Tyrosine phosphorylation profiling of PC12 cells expressing human ALK with and without activation of ALK was undertaken by immunoaffinity purification with P-Tyr-100 (Cell Signaling Technology, Danvers, MA, USA), followed by LC-MS/MS. This was performed as previously described 29–57, except that 20 and 0.8 pmol of the stable isotope-labeled phosphotyrosine peptides EH[13C_3_,15N_1_-A]LLApYTLGVK and HTDDEMTGpYV[13C_3_,15N_1_-A]TR, respectively, were spiked into each cell lysate prior to phosphopeptide purification. Mass spectra of two technical replicates were searched against a rat protein database supplemented with the sequence for human ALK (extracted from UniProtKB/TrEMBL ver. 2013_01; 41 749 reviewed and unreviewed sequences) with maxquant version 1.3.0.5. Standard settings were used in maxquant (false discovery rate of < 1% at the protein, peptide and modification site levels), except that the ‘match between runs’ function was enabled. A list with high-confidence phosphorylation site identifications was generated by filtering the ‘Phospho(STY) sites’ output table for Localization probability > 75% and Score difference > 5. Intensity values for phosphorylation sites were normalized between samples by using correction factors based on the intensity values of the phosphotyrosine sites of the heavily labeled peptide standards. Changes in intensity ratios between cells with activated ALK and control cells of ≥ 5 were considered to be significantly altered.

### Network analysis

Rat–human orthologs were extracted from ensembl. The protein–protein interactions among proteins of interest were retrieved from the Protein Interaction Network Analysis platform 58, and substrate–kinase relationships were downloaded from the PhosphoSitePlus database 59. cytoscape 60 was used for visualization of networks.

### Cell culture

Stable PC12 Tet-on clones expressing human pTTP-ALK (wild type) have been described previously 33–56. Stable clones were selected in DMEM containing 10% horse serum (MP Biomedicals, France), 5% tetracycline-screened fetal bovine serum (Thermo Scientific “HyClone”, Belgium), penicillin, streptomycin, l-glutamine, 100 mg·mL^−1^ G418, and 2 mg·mL^−1^ puromycin, at 37 °C and 5% CO_2_. The neuroblastoma cell lines CLB-GA (1p deletion, 11q deletion, 17q gain, ALK^R1275Q^ mutation), CLB-GE (MYCN/ALK-amplified, ALK^F1174V^ mutation, 1p deletion, 17q gain), CLB-BAR (amplified MYCN/ALK^Δexon 4–12^, 1p deletion, 17q gain) and Kelly (MYCN-amplified, ALK^F1174L^ mutation) were maintained in RPMI-1640 medium supplemented with 10% fetal bovine serum (Sigma, Stockholm, Sweden), 100 U·mL^−1^ penicillin, and 100 μg·mL^−1^ streptomycin, at 37 °C in humidified air with 5% CO_2_ 38–40.

### Cell proliferation

CLB-GA (0.035 × 10^6^ per well), CLB-GE (0.025 × 10^6^ per well), CLB-BAR (0.05 × 10^6^ per well) and Kelly (0.035 × 10^6^ per well) cells were seeded in collagen-coated 48-well plates overnight, and, starting on the following day, treated with FLLL32 (1.5 μm) for 0–5 days. Cell viability was determined at 0–5 days after drug treatment, with 55 μm resazurin (Sigma, Stockholm, Sweden) 61. After 2 h at 37 °C, the amount of metabolized resazurin was analyzed as relative fluorescence with an Infinit200 plate reader (TEKAN, Männedorf, Switzerland). Results are from one of three representative experiments, with each experiment being performed in triplicate. Results are presented as fold relative fluorescence from FLLL32-treated cells versus relative fluorescence from control untreated cells.

### Cell lysis and immunoblotting

Cells were harvested and lysed as described previously 33–36. Samples were boiled in SDS/PAGE sample buffer, and analyzed on SDS/PAGE gel. The antibodies used were anti-STAT3 (#9132), anti-p-STAT3 (Tyr705) (#9145), anti-p-ALK (Tyr1278) (#6941), anti-p-ERK (phospho-p44/42) (#9101), anti-N-MYC (#9405) (all Cell Signaling Technology, MA, USA), anti-FLAG (clone M2, #F3165; Sigma, Stockholm, Sweden), anti-ERK (610123; BD Transduction, Stockholm, Sweden), and anti-ALK (mAb135 and mAb31) 10.

### STAT3 siRNA transfection

Cells were transfected with siRNA duplexes STAT3VHS40491 (siRNA #1: sense, 5′-GCAGUUUCUUCAGAGCAGGUAUCUU-3′; antisense, 5′-AAGAUACCUGCUCUGAAGAAACUGC-3′) and STAT3VHS40497 (siRNA #2: sense, 5′-CCUGCAAGAGUCGAAUGAAUGUUCUCUAU-3′; antisense, 5′-AUAGAGAACAUUCGACUCUUGCAGG-3′) with Lipofectamine 2000, according to the manufacturer's instructions (Invitrogen, Carlsbad, CA, USA). After 6 h, the medium was changed to complete medium without antibiotic, and allowed to grow for 24 or 48 h. Cells were collected and processed for immunoblotting as described above to determine the levels of STAT3, MYCN and pan-ERK for a loading control.

### Immunoprecipitation

PC12 cells were transfected with pcDNA3–hALK or pcDNA3–hALKF1174S (0.6 μg per 2 × 10^6^ cells) together with STAT3–FLAG (0.6 μg per 2 × 10^6^ cells) or pcDNA3 (1.2 μg per 2 × 10^6^ cells), with the Amaxa Biosystems (Cologne, Germany) electroporation procedure. Cells were harvested, and cell lysates were pretreated with protein G–Sepharose (Sigma), before being incubated with the anti-FLAG M2-coupled protein G–Sepharose beads overnight at 4 °C, washed five times with NaCl/Tris, boiled in sample loading buffer, run on an SDS/PAGE gel, transferred to poly(vinylidene difluoride) membranes, and probed with antibodies as indicated.

### Luciferase assay

Cells (2 × 10^5^) of the neuroblastoma cell lines CLB-GE and CLB-BAR were transfected with empty pGL2 (control) or *MYCNP*–luciferase, by the use of Lipofectamine 2000 (Invitrogen) according to the manufacturer's protocol. Cells were then serum-starved, and STAT3 was inhibited by employing 2.5 μm FLLL32 (generated in the laboratory of P.-K. Li) or STATTIC (Sigma, St Louis, MO, USA) for 12 h. Each sample within an experiment was analyzed in triplicate, and the experiment was carried out three times. Results are presented as relative luciferase activity, where untreated samples transfected with empty pGL2 vector were set to 1.

### qRT-PCR

The neuroblastoma cell lines CLB-GE and CLB-BAR were starved and treated with 2.5 μm FLLL32 or STATTIC for 12 h. RNA was isolated with the NucleoSpin RNA II Kit (Macherey-Nagel, Duren, Germany). One microgram of total RNA was reverse-transcribed with the iScript cDNA Synthesis Kit (Bio-Rad, Sundbyberg, Sweden). For the PCR amplification in an iCycler iQ5 (Bio-Rad), 25 ng of cDNA was used in a total reaction mixture of 20 μL containing 10 μL of Quantimix Easy SYG Kit (Biotools, Madrid, Spain), 250 nm forward and reverse primers, and 0.08 μL of fluorescein (USB; Affimetrix, Santa Clara, CA, USA). Primers amplifying part of the coding sequence of RPL29 were used to control for differences in cDNA input. The following primers were used: human MYCN (forward, 5′-ACCACAAGGCCCTCAGTACC-3′; reverse, 5′-TCTCCACAGTGACCACGTCGATTT-3′); human RPL19 (forward, 5′-AACACATCCACAAGCTGAAGGCAG-3′; reverse, 5′-TCTTCACGGCGCTTGCGT-3′); and human RPL29 (forward, 5′-ATGGCCAAGTCCAAGAACCACA-3′; reverse, 5′-TTGGCATTGTTGGCCTGCAT-3′). Relative expression was calculated according to the ΔΔCt relative quantification method. Each sample within an experiment was analyzed in duplicate, and the experiment was carried out at least three times. Results are presented as relative *MYCN* mRNA expression where untreated samples were set to 1.

## Abbreviations

ALK: anaplastic lymphoma kinase
EML4: echinoderm microtubule-associated protein-like 4
ERK: extracellular signal-related kinase
MAPK: mitogen-activated protein kinase
MYCN: myelocytomatosis neuroblastom
NPM: nucleophosmin
NSCLC: non-small-cell lung cancer
PARP: poly(ADP-ribose) polymerase
qRT-PCR: quantitative RT-PCR
RPL19: ribosomal protein 19
RPL29: ribosomal protein 29
RTK: receptor tyrosine kinase
siRNA: small interfering RNA
STAT3: signal transducer and activator of transcription 3
