# Supplementary material for: Phosphoproteomic analysis of anaplastic lymphoma kinase (ALK) downstream signaling pathways identifies signal transducer and activator of transcription 3 as a functional target of activated ALK in neuroblastoma cells
Source: FEBS J. 2013 Aug 22;280(21):5269–82. doi: 10.1111/febs.12453 (PMC3892176; doi:10.1111/febs.12453)
Supplement: Supplementary file 1 [file febs0280-5269-sd1.zip › febs12453-sup-0002-FigS1-S3.pdf]

WILEY Blackwell

the FEBS Journal

www.febsjournal.org

Phosphoproteomic analysis of anaplastic lymphoma kinase (ALK) downstream signaling pathways identifies signal transducer and activator of transcription 3 as a functional target of activated ALK in neuroblastoma cells

Kamaraj Sattu, Falko Hochgräfe, Jianmin Wu, Ganesh Umapathy, Christina Schönherr, Kristina Ruuth, Damini Chand, Barbara Witek, James Fuchs, Pui-Kai Li, Fredrik Hugosson, Roger J. Daly, Ruth H. Palmer and Bengt Hallberg

DOI: 10.1111/febs.12453

Supporting information for Sattu, Hochgräfe et al.,

Supplementary Table 1. Phospho-tyrosine profiling of human ALK in PC12 cells.

Supplementary Figure 1. Verification of phosphorylated downstream target by active ALK in neuroblastoma cell lines. Neuroblastoma cell lines CLB-BAR, CLB-GA and CLB-GE were starved overnight followed by treatment with Crizotinib (250nM) for 6 hours. Whole cell lysates were separated with SDS-PAGE followed by immunoblotting using antibodies against (i) ALK, (ii) p-ALK (Y1278), (iii) p-FAK (Y397), (iv) p-STAT3 (Y705), (v) p-GSK3α (S21), (vi) p-ERK1/2 (T202/Y204) and (vii) p-CrkL (Y207). Pan-Erk was used as a loading control.

Supplementary Figure 2. Immunoprecipitation of ALK and STAT3. PC12 cells transfected with either ALK^{WT} or the ALK^{F1174S} mutant together with flag-tagged STAT3 prior to stimulation with 1 μg/ml of ALK-activating monoclonal antibody (mAb 46) for 24 hours, in the presence or absence of 250 μM crizotinib (C) as indicated. Lysates were immunoprecipitated with anti-ALK (mAb 31) antibodies, followed by immunoblotting for ALK, and STAT3 as indicated. Results were quantified from three individual experiments.

Supplementary Figure 3. Endogenous ALK and MYCN expression in employed cell lines. 0.6×10^6 cells/ml of each CLB-BAR, CLB-GA, CLB-GE and Kelly were seeded and starved overnight. Whole cell lysates were separated on SDS-PAGE followed by immunoblotting with antibodies against MYCN and ALK. Third panel from the top is a more exposed MYCN (long exp.) panel than the one above (short exp). Pan-Erk was used as control for equal loading.

Supplementary Figure 1, Sattu et al. 2013

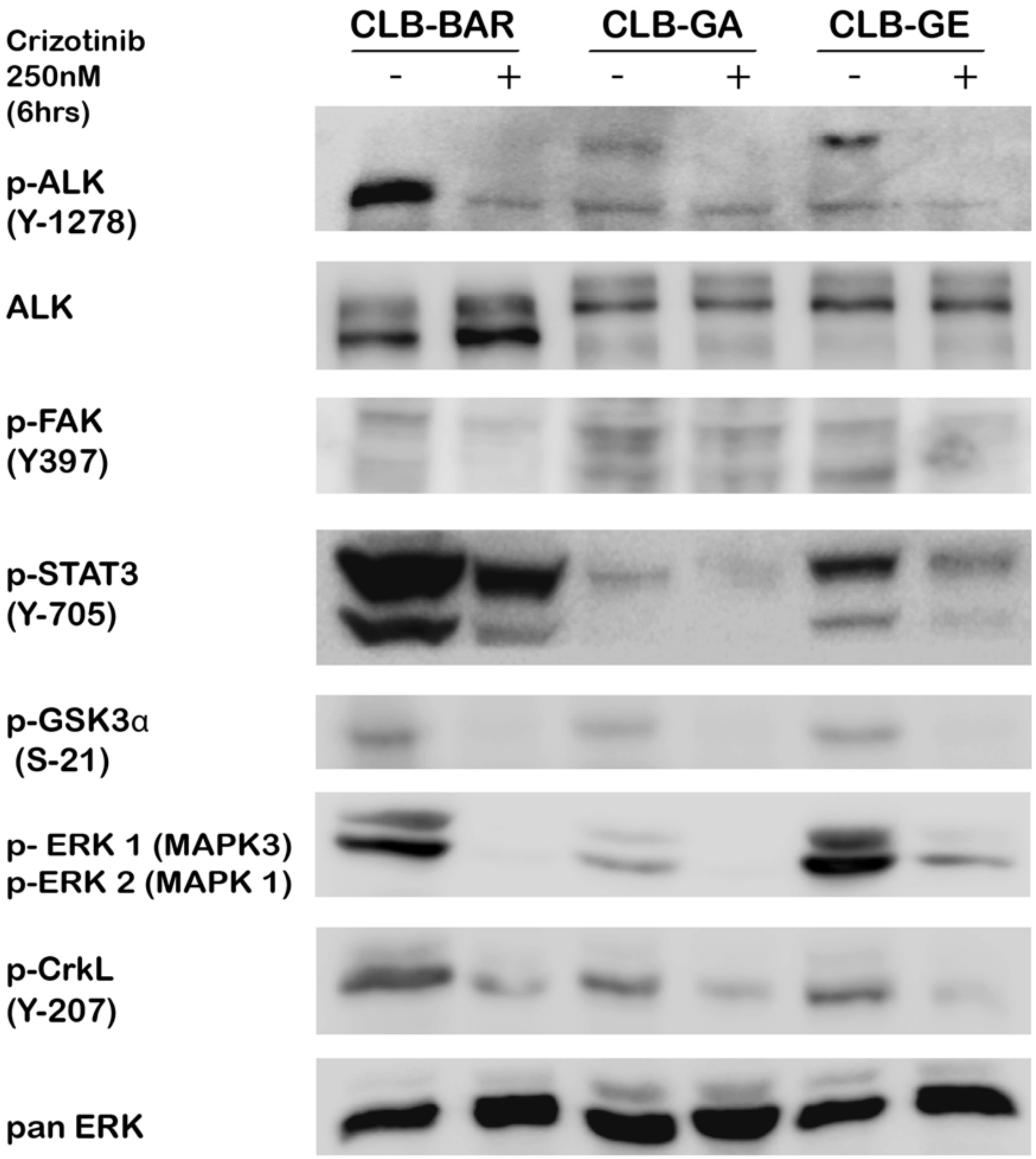

Supplementary Figure 2. Sattu et al, 2013

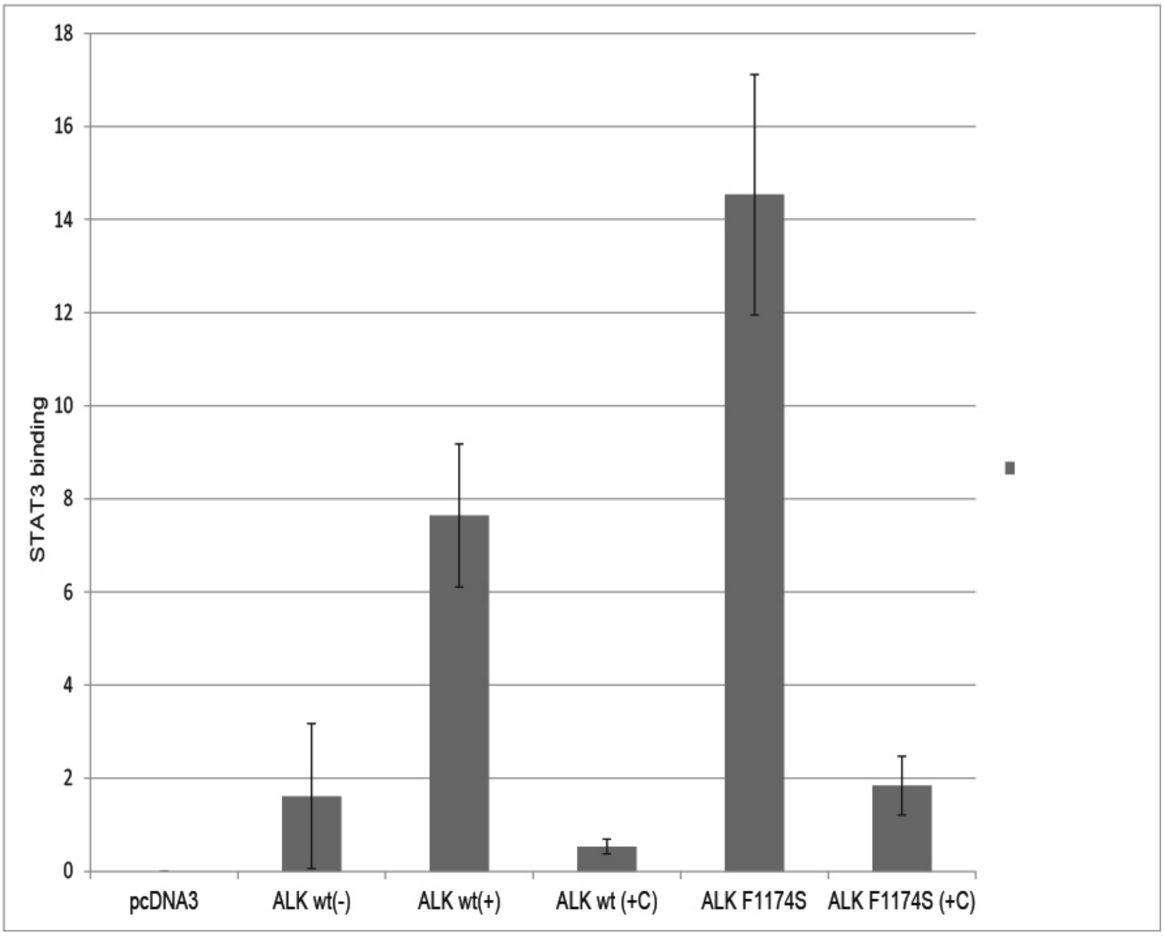

Supplementary figure 3, Sattu et al. 2013

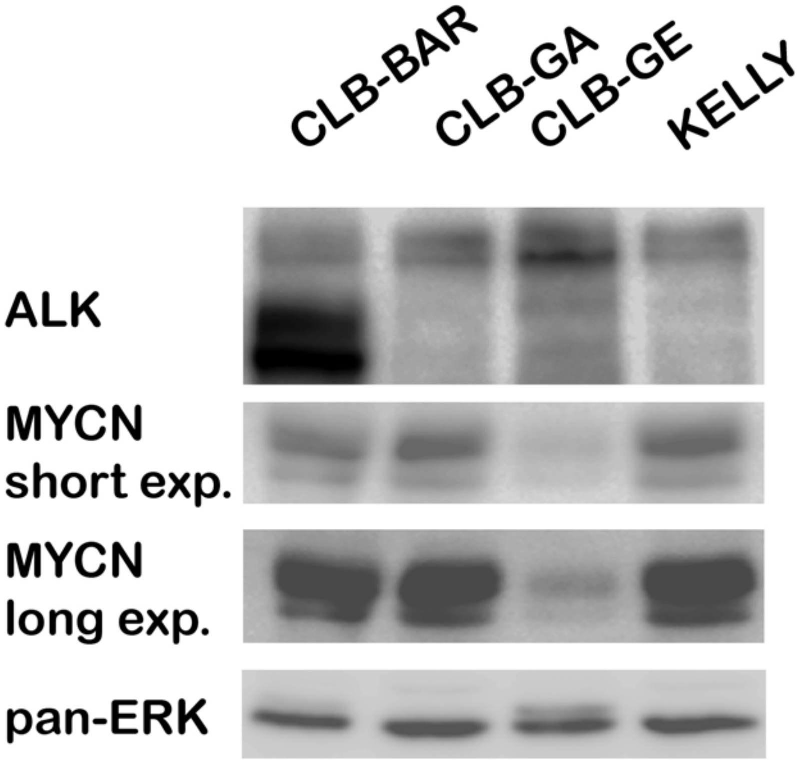

Supplementary Table 1 - Mass spectromtery-based phosphotyrosine profiling in PC12 cells expressing human ALK

Entry	Entry name	Protein na	Gene names	Amino acid	Position	Localization Probability	Score diff	Mean N	Mean N	DELTA log10 hAlk (+Dox vs -Dox)	Sequence W	Modified Sequen	Charge	m/z	Mass error	PEP	Score	Score for localization
Q9UM73	ALK_HUMAN	ALK tyrosir	ALK	Y	1584	0.94	12.03	4.3	7.3	3.0	VPLFRLRHFI	_HFPCGNVNY(ph)	4	994.9405	-0.2829	3.26E-06	90.9	90.9
P18265	GSK3A_RAT	Glycogen s	Gsk3a	S	278	1.00	23.49	5.2	7.7	2.6	DFGSAKQLV	_GEPNVS(ph)YICS	2	681.2814	0.0689	4.29E-11	171.3	71.5
P62944	AP2B1_RAT	AP-2 comp	Ap2b1, Clapb1	Y	277	1.00	30.89	4.3	6.6	2.3	VLMKFLELLI	_DSDYY(ph)NMLL	2	671.2753	0.0042	2.49E-09	172.9	172.9
D4AA64	D4AA64_RAT	Protein Zc	Zc3h7b, Zc3h7b_pr	Y	656	0.96	14.08	4.3	6.6	2.3	FAHSFIELKV	_VWLLQQY(ph)SC	3	824.3819	-0.2687	1.25E-04	126.0	119.5
D3ZWT0	D3ZWT0_RAT	Protein Re	Reps1	Y	126	0.99	19.33	4.3	6.3	2.0	RHAASYSSD	_HAASYSSDAENQ	3	874.7255	0.0139	6.75E-04	79.1	79.1
G3V6D2	G3V6D2_RAT	RCG35087	Socs3, rCG_35087	Y	204	1.00	26.07	4.3	6.2	1.9	LQHLCRKTV	_TVNGHLDSY(ph)	2	671.7874	-0.3738	2.20E-13	175.5	175.5
D3ZF30	D3ZF30_RAT	Protein Cyl	Cyb5rl, rCG_50164	S	256	1.00	50.03	4.3	6.1	1.8	LVARCRRRP	_RPFT(ph)LVCGS(	3	770.9624	0.7166	4.15E-02	50.0	50.0
D3ZF30	D3ZF30_RAT	Protein Cyl	Cyb5rl, rCG_50164	T	251	1.00	50.03	4.3	6.1	1.8	ELVDELVAR	_RPFT(ph)LVCGS(	3	770.9624	0.7166	4.15E-02	50.0	50.0
D3ZF30	D3ZF30_RAT	Protein Cyl	Cyb5rl, rCG_50164	T	260	1.00	50.03	4.3	6.1	1.8	CRRRPFTLV	_RPFT(ph)LVCGS(	3	770.9624	0.7166	4.15E-02	50.0	50.0
Q9UM73	ALK_HUMAN	ALK tyrosir	ALK	Y	1131	1.00	46.56	4.3	6.1	1.8	ITLIRGLGHG	_GLGHGAFGEVY(	3	1040.8214	-0.4420	3.73E-10	121.4	121.4
F1LVS4	F1LVS4_RAT	Protein Ple	Plekha7	T	399	0.82	6.46	4.3	6.1	1.8	RQGPSQPPS	_QGPSQPPSFLEN	2	1069.9990	-0.4556	3.87E-04	123.4	123.4
D3ZCN4	D3ZCN4_RAT	Disks large	Dlg1	Y	783	1.00	44.67	4.3	6.1	1.8	HTTRPKRDY	_RDYEVDGRDY(pl	3	698.9725	-0.1129	9.65E-07	139.6	127.3
Q63803	GNAS1_RAT	Guanine n	Gnas, Gnas1	Y	579	1.00	176.83	4.3	6.1	1.8	SAMSAAAR	_AAY(ph)AGPLVW	2	656.3159	0.0626	9.79E-16	176.8	176.8
D3ZQ60	D3ZQ60_RAT	Protein Ple	Plekhg4	T	431	1.00	81.53	4.3	6.0	1.7	ADELYGQVI	_ADELY(ph)GQVD	3	758.3587	1.4750	2.55E-04	95.1	81.5
D3ZQ60	D3ZQ60_RAT	Protein Ple	Plekhg4	Y	420	1.00	81.53	4.3	6.0	1.7	LSPDYRSAV	_ADELY(ph)GQVD	3	758.3587	1.4750	2.55E-04	95.1	81.5
G3V9D7	G3V9D7_RAT	Adducin 3	Add3, rCG_57421	Y	446	0.84	7.21	4.3	6.0	1.7	RQQREKTR	_WLNSPNTY(ph)N	2	667.2860	0.0410	2.03E-06	136.0	136.0
Q07936	ANXA2_RAT	Annexin A	Anxa2, Anx2	Y	316	0.98	16.32	4.3	6.0	1.7	LKIRSEFKRK	_SLY(ph)YFIQQDT	2	743.3367	0.1983	2.15E-11	158.4	158.4
Q9UM73	ALK_HUMAN	ALK tyrosir	ALK	Y	1092	1.00	36.14	4.3	6.0	1.6	EYKLSKLRTS	_TSTIMTDY(ph)NI	2	1031.9179	0.2394	7.95E-20	203.0	203.0
Q5BK33	Q5BK33_RAT	p55 protei	LOC652956	Y	332	1.00	45.98	4.3	5.9	1.6	HTTRPPRKG	_KGEADGAEY(ph)	3	726.3050	-0.0674	2.99E-04	122.1	122.1
Q63487	RRAGB_RAT	Ras-relate	RragB	S	309	1.00	27.89	4.3	5.9	1.6	NIIKQFKLSC	_LS(ph)CSKLAAS(	2	1035.3973	-0.2516	1.30E-02	69.0	62.7
Q63487	RRAGB_RAT	Ras-relate	RragB	S	312	1.00	21.99	4.3	5.9	1.6	KQFKLSCSKI	_LS(ph)CSKLAAS(	2	1035.3973	-0.2516	1.30E-02	69.0	62.7
D3ZW17	D3ZW17_RAT	Protein Slc	Slc1a5, rCG_53545	T	528	1.00	67.33	4.3	5.9	1.6	IQVKNDVSL	_PLPLAT(ph)EEGN	2	786.4077	-0.2563	1.76E-02	67.3	67.3
P18265	GSK3A_RAT	Glycogen s	Gsk3a	S	282	1.00	44.70	4.3	5.8	1.5	AKQLVRGEI	_GEPNVSY(ph)ICS	2	721.2646	-0.0698	3.17E-03	80.5	80.5
F1M2K6	F1M2K6_RAT	Uncharacterized protein (Fragi		Y	426	1.00	41.57	4.3	5.7	1.4	RTSSQRSTL	_NNY(ph)ALNTAA	3	852.3953	0.4545	1.08E-02	81.7	81.7
D4AAT7	NNRD_RAT	ATP-deper	Carkd	Y	81	0.78	5.59	4.3	5.7	1.4	KGQDGRIGI	_IGIVGGCQEY(ph)	3	818.0588	-0.4311	3.73E-04	89.0	89.0
Q9UM73	ALK_HUMAN	ALK tyrosir	ALK	S	1281	1.00	29.02	5.5	6.9	1.4	AKIGDFGM	_DIY(ph)RAS(ph)Y	2	723.7482	-0.0478	6.41E-10	176.8	176.8
D3ZME6	D3ZME6_RAT	Heterogen	Syncrip	Y	373	1.00	84.61	4.3	5.7	1.4	FSQFGKLER	_LKDY(ph)AFIHFD	3	545.2519	-0.4516	1.28E-02	84.6	84.6
Q9UM73	ALK_HUMAN	ALK tyrosir	ALK	Y	1078	1.00	69.86	6.0	7.3	1.4	HQELQAMO	_HQELQAMQMEL	2	1085.4709	-0.3678	1.69E-13	191.5	168.1
Q9UM73	ALK_HUMAN	ALK tyrosir	ALK	Y	1283	1.00	41.80	5.7	7.1	1.3	IGDFGMARI	_DIY(ph)RAS(ph)Y	2	723.7482	-0.0478	6.41E-10	176.8	176.8
D3ZSM1	D3ZSM1_RAT	Protein Ep	Epb4.1l2, RGD1563	Y	615	1.00	177.06	4.3	5.6	1.3	AEGKKNTLF	_VDGDNIY(ph)VR	2	565.7475	0.0612	1.97E-10	177.1	177.1
P05370	G6PD_RAT	Glucose-6-	G6pdx, G6pd	T	402	0.75	4.85	4.3	5.6	1.3	RNELVIRVQ	_VQPNEAVYT(ph)	2	614.7841	-0.0498	1.05E-02	83.9	72.0
Q9UM73	ALK_HUMAN	ALK tyrosir	ALK	Y	1278	1.00	60.23	5.9	7.2	1.3	GRVAKIGDF	_DIY(ph)RAS(ph)Y	2	723.7482	-0.0478	6.41E-10	176.8	176.8
Q9JJ50	HGS_RAT	Hepatocyt	Hgs, Hrs, Hrs2	Y	132	0.86	7.95	4.3	5.6	1.3	HAFRNEPKY	_VVQDTY(ph)QIM	2	652.8015	0.3657	1.19E-06	143.5	113.9
Q9UM73	ALK_HUMAN	ALK tyrosir	ALK	Y	1282	0.96	13.55	5.3	6.6	1.3	KIGDFGMAI	_DIY(ph)RASY(ph)	3	525.5329	-0.0903	6.24E-03	94.3	94.3
Q9JJ50	HGS_RAT	Hepatocyt	Hgs, Hrs, Hrs2	T	131	0.96	13.90	4.3	5.6	1.3	AHAFRNEPI	_YKVVQDT(ph)YC	2	798.3806	0.7521	4.75E-03	101.5	89.5
Q9UM73	ALK_HUMAN	ALK tyrosir	ALK	Y	1604	0.99	18.37	5.9	7.1	1.3	QGLPLEAAT	_HFPCGNVNYGY(	3	1326.2516	-0.3040	4.38E-17	144.2	144.2
Q9UM73	ALK_HUMAN	ALK tyrosir	ALK	Y	1096	1.00	78.54	6.2	7.5	1.2	SKLRTSTIM	_LRTS(ph)TIMTDY	3	804.6649	0.0358	1.38E-49	278.3	123.1
P62828	RAN_RAT	GTP-bindir	Ran	Y	147	0.82	6.77	4.3	5.5	1.2	KAKSIVFHRI	_NLQYY(ph)DISAK	2	647.7894	0.5200	1.88E-02	73.9	73.9
F1LQ24	F1LQ24_RAT	Rho GTPas	Arhgap27	Y	227	1.00	40.86	4.3	5.5	1.2	RAKSPKQVI	_QVDEPPEPVY(ph	2	911.4064	-0.2089	5.01E-03	80.4	40.9
D4A8H8	D4A8H8_RAT	Cytoplasm	Cyfip1, Cyfip1_prec	S	556	0.78	9.16	4.3	5.5	1.2	SGFDIKVPRI	_AVGPSS(ph)TQL	2	744.8495	-0.0670	4.43E-02	78.8	78.8
G3V913	G3V913_RAT	Heat shock	Hspb1, rCG_21815	Y	137	1.00	25.17	4.3	5.5	1.2	VEITGKHEEI	_QDEHGY(ph)ISR_	2	592.7402	0.2919	9.63E-03	80.2	80.2
D4A563	D4A563_RAT	Protein RG	RGD1312026, RGD1	Y	637	1.00	60.03	4.3	5.5	1.2	VPIVINPNA	_NAIKVPIVINPNA	3	835.0848	-0.1426	1.61E-02	60.0	60.0
F8WFW8	F8WFW8_RAT	Mitogen-a	Mapk1	T	183	1.00	67.34	5.5	6.6	1.2	LARVADPDI	_VADPDHDHTGFL	3	768.6505	0.0469	8.83E-09	168.8	168.8
Q9UM73	ALK_HUMAN	ALK tyrosir	ALK	T	1597	0.91	11.61	6.3	7.4	1.1	VNYGYQQC	_HFPCGNVNYGYC	4	974.9489	-0.3453	4.24E-06	90.9	72.6
Q9UM73	ALK_HUMAN	ALK tyrosir	ALK	S	1086	0.89	11.85	5.2	6.3	1.1	MELQSPEYI	_LRTS(ph)TIMTDY	2	1206.4937	-0.0093	5.59E-19	207.1	204.1
G3V696	G3V696_RAT	BRCA1-A c	Fam175a, RGD1305	T	169	1.00	55.58	4.3	5.4	1.1	YKPQSGLFH	_VPLVVT(ph)NLGI	3	643.9846	0.0304	1.98E-02	77.4	77.4
D3ZL58	D3ZL58_RAT	Protein Nu	Nub1	Y	127	1.00	91.50	5.0	6.1	1.1	DLRSKIAETF	_IAETFGFQENY(pl	2	820.3738	-0.2299	8.88E-33	235.3	185.0
D3ZEN7	D3ZEN7_RAT	Uncharacterized protein		Y	12	1.00	103.70	4.3	5.4	1.1	____MLVPI	_IAIY(ph)ELLFK_	2	595.3171	0.4426	2.13E-03	103.7	103.7
Q9UM73	ALK_HUMAN	ALK tyrosir	ALK	Y	1586	0.99	20.73	6.1	7.2	1.1	LFRLRHFPC	_HFPCGNVNYGY(	4	994.9405	-0.3534	4.38E-17	144.2	100.2
D3ZN31	D3ZN31_RAT	Protein RG	RGD1565350	Y	260	0.99	19.41	4.3	5.4	1.1	KNDLKSKAG	_AGKGESAGY(ph)	3	641.9363	-0.1425	4.16E-04	121.8	121.8
D3ZIT3	D3ZIT3_RAT	Protein Nir	Nin	T	1881	1.00	35.42	4.3	5.3	1.0	HSREKIRQLI	_IRQLESNLLPT(ph	2	746.4002	5.5316	5.99E-02	83.0	83.0
P41499	PTN11_RAT	Tyrosine-p	Ptpn11	Y	546	0.89	8.99	4.3	5.3	1.0	RRIEEEQKSI	_KGHEY(ph)TNIK_	2	585.2712	-0.0094	2.03E-22	232.4	119.8
E9PTU7	E9PTU7_RAT	Protein R3	R3hdm2	S	380	0.87	7.05	4.3	5.3	1.0	SDGSVRSM	_AS(ph)S(ph)FSGI	3	493.5292	0.5636	4.11E-02	37.0	37.0
E9PTU7	E9PTU7_RAT	Protein R3	R3hdm2	S	381	0.87	7.05	4.3	5.3	1.0	DGSVRSMR	_AS(ph)S(ph)FSGI	3	493.5292	0.5636	4.11E-02	37.0	37.0
E9PTU7	E9PTU7_RAT	Protein R3	R3hdm2	S	386	0.88	7.64	4.3	5.3	1.0	SMRPPVTK	_AS(ph)S(ph)FSGI	3	493.5292	0.5636	4.11E-02	37.0	37.0
P41499	PTN11_RAT	Tyrosine-p	Ptpn11	Y	584	1.00	187.44	5.0	6.0	1.0	PTPPCAEMI	_VY(ph)ENVGLMC	2	772.8500	0.3091	3.87E-16	189.2	187.4
Q4V8A3	DYRK3_RAT	Dual speci	Dyrk3	Y	368	1.00	24.91	4.3	5.3	1.0	IDFGSSCFEY	_LYTY(ph)IQSR_	2	562.2628	-0.1172	2.17E-04	134.3	124.9
Q9UM73	ALK_HUMAN	ALK tyrosir	ALK	Y	1507	0.98	16.65	6.2	7.2	1.0	VHGSRNKPT	_NKPTSLWNPTY(	3	854.7403	0.0758	1.09E-18	205.1	205.1
P52631	STAT3_RAT	Signal tran	Stat3	Y	705	1.00	51.92	5.6	6.7	1.0	ESQEHPEAD	_YCRPESQEHPEAI	3	861.3721	-0.1364	9.51E-13	183.2	183.2
F1LVS4	F1LVS4_RAT	Protein Ple	Plekha7	T	582	0.76	5.09	4.3	5.3	1.0	HTVSAPSLH	_SADDT(ph)YLQLI	2	681.3211	0.2247	5.33E-11	161.7	161.7
Q9Z1T4	CNKR2_RAT	Connector	Cnksr2	Y	821	0.97	15.87	4.3	5.3	1.0	RQSTLPTQK	_CHLQDHY(ph)GF	3	717.9700	0.7763	2.23E-02	58.8	58.8
Q5RJR2	TWF1_RAT	Twinfilin-1	Twf1, Ptk9	Y	309	1.00	98.90	5.3	6.3	1.0	KIEIDNGDEI	_KIEIDNGDELTAD	3	881.0738	-0.1895	2.42E-19	203.3	160.7
O35889	AFAD_RAT	Afadin (Prc	Mllt4, Af6	Y	263	1.00	85.14	5.6	6.6	1.0	GTLRIYADSI	_IYADSLKPNIPY(p	2	801.4024	-0.1541	1.70E-17	186.8	164.5
F1LN42	F1LN42_RAT	Protein Tn	Tns1	Y	1336	0.76	5.26	4.3	5.3	1.0	VPGSPSLDR	_HVAYGGY(ph)ST	2	766.3143	0.4345	1.44E-05	122.5	122.5
F1LQ91	F1LQ91_RAT	Protein Do	Dock11	Y	23	0.95	12.77	4.3	5.3	1.0	PLDYENVITC	_KTQIY(ph)SDPLR	2	650.8185	-0.8987	1.67E-02	70.8	70.8
Q6P767	PTTG_RAT	Pituitary tu	Pttg1ip	Y	171	1.00	171.73	4.3	5.2	0.9	DEIRKKYGLI	_EQNPY(ph)EKF_	2	567.7288	-0.0153	4.69E-07	171.7	171.7
D3ZB32	D3ZB32_RAT	Protein Ep	Epb41l3	Y	554	1.00	114.40	5.3	6.2	0.9	ATENSLIKRI	_IKGENVY(ph)VK_	2	565.2863	-0.0141	1.15E-03	126.3	114.4
Q5U302	Q5U302_RAT	Catenin (C	Ctnna1, Catna1, rCC	Y	282	0.99	23.16	5.3	6.2	0.9	ASQQQGGC	_QLQQAVTGISNA	4	1072.9899	-0.4194	6.82E-04	65.5	65.5
P41499	PTN11_RAT	Tyrosine-p	Ptpn11	T	547	0.75	4.85	4.3	5.2	0.9	RIEEEQKSKI	_KGHEYT(ph)NIK_	2	585.2712	-0.0794	1.52E-04	140.5	93.3
D3ZIA3	D3ZIA3_RAT	LIM and SH	Lasp1	Y	57	1.00	33.05	4.3	5.2	0.9	KNYKGYEKK	_KPYCNAHY(ph)P	2	679.2916	0.0408	1.61E-02	71.6	71.6
Q6TXF3	Q6TXF3_RAT	LRRGT0004	Dbi, LOC679040	Y	128	0.92	10.74	5.5	6.4	0.9	KRLKTQPTD	_LKTQPTDEEMLFI	3	769.6971	0.1300	1.12E-13	195.9	195.9
Q9Z1X1	ESYT1_RAT	Extended s	Esyt1, Fam62a, Mb	Y	351	1.00	52.74	5.2	6.1	0.9	KDKYVKGLII	_SDPY(ph)ALVR_	2	500.7286	-0.3515	2.06E-03	117.4	86.8
Q9Z1X1	ESYT1_RAT	Extended s	Esyt1, Fam62a, Mb	Y	811	0.98	16.39	6.2	7.1	0.9	SEDLPLRKG	_GTKPPSPY(ph)AI	3	688.3452	-0.1689	2.13E-20	218.7	170.1
G3V9T9	G3V9T9_RAT	Protein tyr	Ptpn6, rCG_29721	Y	566	1.00	103.77	5.2	6.1	0.9	HAKASRTSS	_HKEEVY(ph)ENV	3	526.9031	0.0543	2.68E-20	200.2	181.7
G3V9E1	G3V9E1_RAT	Protein kin	Prkacb, Prkacb_pre	Y	117	0.88	6.30	4.3	5.2	0.9	FGRVMLVKI	_HKATEQY(ph)Y(	2	765.2984	0.1613	2.25E-02	78.7	44.3
D4A446	D4A446_RAT	Protein LO	LOC689959, rCG_5	Y	54	1.00	32.27	4.3	5.1	0.8	LWQNGTKD	_DYETTAPMY(ph)	2	728.2785	0.1721	6.09E-03	78.3	78.3
F1M7A0	F1M7A0_RAT	DNA topoi	Top2a	S	810	1.00	23.27	4.3	5.1	0.8	GGKDSASPI	_Y(ph)IFTMLS(ph)	3	491.2201	-0.2169	6.68E-02	47.6	47.6
D4A244	D4A244_RAT	Fibroblast	Frs2, Frs2_predicte	Y	349	1.00	33.52	4.6	5.4	0.8	QNINNSAQI	_RPALLNY(ph)ENI	3	805.7455	-0.3705	4.80E-03	79.7	79.7
B0K008	B0K008_RAT	Eukaryotic	Eif1, rCG_33041	Y	30	1.00	36.66	5.3	6.2	0.8	DASKGDDLI	_GDDLLPAGTEDY	2	932.9275	-0.4115	3.74E-23	218.7	218.7
Q156J1	Q156J1_RAT	Bcl-2-inter	Bag3	Y	250	1.00	86.68	5.5	6.2	0.8	HYPAQQGE	_THYPAQQGEYQI	3	793.6900	0.2811	2.48E-06	150.8	128.7
B5DEX4	B5DEX4_RAT	Protein Va	Vasp	Y	40	1.00	89.45	5.9	6.6	0.8	LPAGTGPQ	_VQIY(ph)HNPTAI	2	813.8749	-0.0054	4.55E-63	292.6	292.6
D3ZCE5	D3ZCE5_RAT	Protein Gp	Gpsm2	Y	146	1.00	169.15	5.9	6.7	0.8	ISRELNDKV	_ALY(ph)NLGNVY	3	481.5658	-0.3506	9.29E-28	223.1	215.9
G3V8F6	G3V8F6_RAT	G-protein	Gpsm1, rCG_45366	Y	340	1.00	94.41	5.2	6.0	0.8	GNTYTLLQD	_AAEY(ph)HLR_	2	470.2078	0.1664	2.21E-04	141.0	94.4
G3V9T9	G3V9T9_RAT	Protein tyr	Ptpn6, rCG_29721	Y	538	0.95	13.20	5.6	6.3	0.8	KKLEIIQSQR	_GQESEY(ph)GNI	2	937.9197	0.2714	8.71E-11	169.4	169.4
P21708	MK03_RAT	Mitogen-a	Mapk3, Erk1, Prkm	T	203	1.00	69.21	6.0	6.7	0.8	LARIADPEH	_IADPEHDHTGFLT	3	777.9943	0.1835	6.85E-13	185.0	150.0
P04764	ENOA_RAT	Alpha-eno	Eno1, Eno-1	Y	44	1.00	33.72	5.7	6.4	0.8	GLFRAAVPS	_AAVPSGASTGIY(	2	942.9588	-0.6673	1.12E-19	207.5	79.8
B2B9A9	B2B9A9_RAT	Ephrin B2	Efnb2, Efnb2_predi	Y	307	1.00	85.08	5.0	5.7	0.8	VIIPLRTADS	_TADSVFCPHY(ph	2	767.3076	-0.0649	4.08E-12	169.0	147.2
Q5XFX0	TAGL2_RAT	Transgelin	Tagln2	Y	192	0.98	17.18	4.3	5.1	0.8	QMGSNKGA	_GASQAGMTGY(	2	732.2940	0.0386	9.52E-09	138.0	138.0
D3ZN29	D3ZN29_RAT	Protein Ah	Ahnak	Y	122	1.00	93.96	5.3	6.0	0.8	SSRSSEVVLS	_SSEVVLSGDDED	2	889.8595	0.4421	8.72E-40	245.4	245.4
Q2HWF0	FBP1L_RAT	Formin-bir	Fnbp1l, Toca1	Y	291	0.95	15.62	5.8	6.5	0.7	EPPGDFPFE	_SGFEPPGDFPFED	3	823.3459	-0.2102	1.55E-04	124.3	124.3
A2RRT6	A2RRT6_RAT	RCG27903	Zc3hav1, rCG_2790	Y	501	0.88	8.48	4.7	5.4	0.7	GFAIKPAIAC	_EAVY(ph)SGVQS	2	636.7846	0.1072	9.49E-03	70.9	67.2
D3ZAZ0	D3ZAZ0_RAT	Protein Eif	Eif3m, RGD156584	T	242	1.00	48.66	4.3	5.0	0.7	KPVKFLEGE	_FLEGELIHDLLT(p	2	1103.0358	0.0910	4.91E-02	57.5	48.7
D3ZAZ0	D3ZAZ0_RAT	Protein Eif	Eif3m, RGD156584	S	246	1.00	48.66	4.3	5.0	0.7	FLEGELIHDI	_FLEGELIHDLLT(p	2	1103.0358	0.0910	4.91E-02	57.5	48.7
Q07936	ANXA2_RAT	Annexin A	Anxa2, Anx2	Y	24	0.99	20.95	6.7	7.4	0.7	CKLSLEGDH	_LSLEGDHSTPPSA	4	755.3434	0.0308	6.32E-13	158.8	96.0
B5DEN4	B5DEN4_RAT	L-lactate d	Ldha	Y	239	1.00	27.22	6.8	7.5	0.7	KEQWKDVH	_QVVDSAY(ph)EV	2	665.8182	-0.0703	1.65E-11	162.4	74.0
Q9Z340	PARD3_RAT	Partitionin	Pard3, Par3	Y	489	0.99	19.65	5.5	6.2	0.7	SITSRDVTIG	_DVTIGGSAPIY(ph	2	700.3471	-0.1980	5.45E-25	203.6	203.6
F1LPK6	F1LPK6_RAT	Protein Ptp	Ptpn18	Y	382	0.99	21.65	5.2	5.9	0.7	TGPGTRAPN	_APNSTDTPIY(ph)	2	898.9144	-0.0016	5.62E-30	233.4	233.4
P11980	KPYM_RAT	Pyruvate k	Pkm, Pkm2, Pykm	Y	390	1.00	87.08	5.1	5.8	0.7	VRMQHLIAI	_EAEAAIY(ph)HLC	3	723.3561	0.2314	1.51E-03	87.1	87.1
D3ZUD6	D3ZUD6_RAT	Focal adhe	Ptk2	Y	611	1.00	47.14	5.6	6.3	0.7	LGDFGLSRY	_YMEDSTY(ph)Y(	2	823.2983	0.0648	2.86E-21	208.3	203.1
Q9WVR3	SHIP2_RAT	Phosphatic	Inppl1, Ship2	Y	887	1.00	57.28	5.5	6.1	0.7	KVRVPTERL	_ERLY(ph)EWISID	3	706.9963	-0.0412	2.49E-05	138.7	138.7
G3V9E1	G3V9E1_RAT	Protein kin	Prkacb, Prkacb_pre	Y	116	0.94	11.99	5.5	6.1	0.7	SFGRVMLVI	_HKATEQY(ph)YA	2	725.3153	0.0576	1.90E-04	113.4	113.4
D4A6D6	D4A6D6_RAT	Protein kin	Strada, Lyk5, rCG_3	S	216	1.00	32.25	4.3	4.9	0.6	ILISTDGKVY	_S(ph)NLS(ph)MIS	2	735.2559	0.2158	4.72E-02	32.3	32.3
D4A6D6	D4A6D6_RAT	Protein kin	Strada, Lyk5, rCG_3	S	219	1.00	32.25	4.3	4.9	0.6	STDGKVYLS	_S(ph)NLS(ph)MIS	2	735.2559	0.2158	4.72E-02	32.3	32.3
D4A6D6	D4A6D6_RAT	Protein kin	Strada, Lyk5, rCG_3	S	222	1.00	32.25	4.3	4.9	0.6	GKVYLSGLR	_S(ph)NLS(ph)MIS	2	735.2559	0.2158	4.72E-02	32.3	32.3
D4ADE8	D4ADE8_RAT	Protein Dd	Ddx3x	Y	104	1.00	118.87	4.8	5.5	0.6	GSGSRGRFI	_GRGDY(ph)DGIG	2	601.7511	-0.1830	1.76E-04	118.9	118.9
B2B9A9	B2B9A9_RAT	Ephrin B2	Efnb2, Efnb2_predi	Y	333	0.78	6.61	5.4	6.0	0.6	VYIVQEMPI	_VSGDYGHPVYIV(	3	1044.4910	-0.5165	1.96E-04	84.2	84.2
D3ZX40	D3ZX40_RAT	Protein Fa	Fam65c	T	18	1.00	42.13	5.3	5.9	0.6	VRLRFLSPGI	_FLSPGEAGAVGT(	2	749.3585	-0.0359	5.79E-02	54.8	54.8
Q07936	ANXA2_RAT	Annexin A	Anxa2, Anx2	Y	238	1.00	51.03	6.3	6.9	0.6	CHLQKVFER	_SYSPY(ph)DMLE	2	770.8231	0.3152	5.78E-16	185.3	119.4
D4A244	D4A244_RAT	Fibroblast	Frs2, Frs2_predicte	Y	306	1.00	88.23	5.5	6.1	0.6	DSDERRDVI	_LVY(ph)ENINGLS	2	984.9932	0.2011	1.99E-38	261.2	224.9
Q66X93	SND1_RAT	Staphylocc	Snd1	Y	108	1.00	45.76	5.4	6.0	0.6	EVCFTIENKT	_EY(ph)GMIYLGK_	2	577.2536	-0.0491	5.45E-03	87.6	87.6
B2GV73	B2GV73_RAT	Actin relat	Arpc3, Arpc3_predi	Y	47	0.91	9.81	4.3	4.9	0.6	APRETKDTD	_DTDIVDEAIY(ph)	2	836.3631	0.2544	8.23E-03	72.7	72.7
D4A6K7	D4A6K7_RAT	Protein Lrr	Lrrc19, rCG_53420	T	24	0.79	7.31	4.3	4.9	0.6	WLFSMLLSI	_SQASET(ph)EVK_	2	529.7237	-0.6835	3.58E-02	67.7	67.7
D3ZQJ5	D3ZQJ5_RAT	Protein kin	Prkcd	Y	311	1.00	73.43	5.4	5.9	0.6	QKASRKPET	_KPETPETVGIY(ph	3	677.6660	-0.2668	1.90E-05	142.3	139.5
P62944	AP2B1_RAT	AP-2 comp	Ap2b1, Clapb1	Y	276	1.00	36.39	5.9	6.5	0.5	KVLMKFLEL	_DSDY(ph)YNMLL	2	735.3227	0.1230	1.54E-27	239.9	235.0
Q63470	DYR1A_RAT	Dual speci	Dyrk1a, Dyrk	Y	145	0.99	22.95	5.3	5.8	0.5	KKERKVYND	_KVYNDGYDDDN	2	1089.9488	0.3035	1.33E-08	154.4	154.4
G3V982	G3V982_RAT	Engulfmen	Elmo2, rCG_32210	Y	48	1.00	28.04	5.4	5.9	0.5	VIKEVCDGV	_EVCDGWSLPNPE	2	1089.9637	0.0220	8.64E-09	155.9	155.9
D3ZUR9	D3ZUR9_RAT	Leucyl-cyst	Lnpep	Y	71	1.00	40.76	4.8	5.3	0.5	VRGLGEHEI	_GLGEHEMDEDEE	3	750.6070	-0.2490	8.80E-04	101.0	101.0
D4A4T4	D4A4T4_RAT	Protein Pvi	Pvrl3, rCG_52915	Y	511	0.97	15.60	5.6	6.1	0.5	NNVENLTRI	_FERPMDYY(ph)E	3	562.5688	0.0558	2.02E-15	186.2	80.2
Q3KRF2	Q3KRF2_RAT	High densi	Hdlbp, rCG_55574	Y	437	1.00	143.24	6.1	6.6	0.5	EQIEGMVKI	_MDY(ph)VEINIDI	3	486.2108	-0.4444	5.27E-72	194.4	143.2
D4A446	D4A446_RAT	Protein LO	LOC689959, rCG_5	Y	106	1.00	34.34	5.2	5.7	0.5	HSLKVKCLR	_SESATEY(ph)ATL	2	654.2794	0.1320	5.37E-16	194.2	194.2
D3ZV15	D3ZV15_RAT	Protein Cb	Cbl	Y	673	0.92	12.48	5.8	6.3	0.5	SEHPKIKPSS	_IKPSSSANAIY(ph	3	787.4294	0.3118	4.07E-03	89.5	89.5
Q4QQV2	DOK1_RAT	Docking pr	Dok1	Y	448	1.00	37.31	5.6	6.1	0.5	GTTAGSGSK	_GSDTALY(ph)SQ	2	688.8083	-0.2101	2.46E-28	236.1	218.6
P05370	G6PD_RAT	Glucose-6-	G6pdx, G6pd	Y	401	0.92	10.74	5.3	5.7	0.4	KRNELVIRV(	_VQPNEAVY(ph)T	2	614.7841	-0.0222	8.52E-03	76.9	76.9
D3ZN31	D3ZN31_RAT	Protein RG	RGD1565350	Y	113	1.00	124.10	4.8	5.2	0.4	PGSSLRKLR	_LDY(ph)CGGGGD	2	773.2930	0.3626	3.71E-06	124.1	124.1
P63255	CRIP1_RAT	Cysteine-ri	Crip1, Crip	Y	12	1.00	91.94	5.4	5.8	0.4	____MPKCI	_CDKEVY(ph)FAEI	2	698.7838	-0.3138	7.44E-03	91.9	91.9
E7CXR8	E7CXR8_RAT	Epidermal	Egfr	Y	1109	1.00	50.06	6.1	6.5	0.4	QSVPKRPAC	_RPAGSVQNPVY(	3	824.7397	0.0305	3.24E-09	171.8	171.8
G3V8S2	G3V8S2_RAT	SHC-transf	Shc1, rCG_62677	Y	349	1.00	36.30	6.3	6.7	0.4	GSAWDEEE	_MAGFDGSAWDE	4	1143.4603	-0.3560	5.83E-04	68.7	68.7
G3V8S2	G3V8S2_RAT	SHC-transf	Shc1, rCG_62677	Y	350	1.00	36.30	6.3	6.7	0.4	SAWDEEEEI	_MAGFDGSAWDE	4	1143.4603	-0.3560	5.83E-04	68.7	68.7
F1LXF1	F1LXF1_RAT	Protein Bc	Bcr	Y	68	1.00	65.92	6.1	6.5	0.4	KGPAQPGS	_KGPAQPGSADAE	4	723.3382	0.0010	6.70E-06	124.8	124.8
F1M2Y4	F1M2Y4_RAT	Protein Slc	Slc29a4	Y	267	1.00	23.78	5.3	5.6	0.4	KDSPAHEVT	_DSPAHEVTHSNS	3	656.2753	-0.4729	3.13E-04	106.4	101.5
D3ZN31	D3ZN31_RAT	Protein RG	RGD1565350	Y	238	0.97	15.23	5.0	5.3	0.4	ETGAGQKD	_DKVTIADDY(ph)	2	940.4035	-0.3893	9.64E-15	186.9	186.9
Q07936	ANXA2_RAT	Annexin A	Anxa2, Anx2	S	22	0.81	6.91	7.1	7.4	0.4	ILCKLSLEGD	_LSLEGDHSTPPS(	4	1022.4888	0.0433	5.21E-10	150.1	82.5
B6ID02	B6ID02_RAT	G protein-	Gprc5c, rCG_34127	Y	386	1.00	59.01	5.4	5.7	0.3	PTEMALMH	_GPSEGAY(ph)DV	2	727.3398	-0.0367	2.41E-12	165.6	165.6
G3V888	G3V888_RAT	ATP citrate	Acly, rCG_33241	Y	682	1.00	50.04	6.2	6.5	0.3	SNELNNIISR	_TTDGVY(ph)EGV	3	998.4548	0.3687	1.80E-10	143.4	138.5
F8WFS9	F8WFS9_RAT	Beta-addu	Add2	Y	489	0.85	7.50	5.0	5.4	0.3	GMPIRIENP	_IENPNQFVPLY(pl	3	866.7364	-0.6275	5.12E-03	69.1	69.1
D3ZQ98	D3ZQ98_RAT	Dihydropy	Crmp1	Y	618	1.00	76.52	5.9	6.3	0.3	FGLHSVSRG	_GMYDGPVY(ph)I	2	852.3730	0.0123	3.08E-30	232.5	232.5
F8WG07	F8WG07_RAT	Calmodulir	Calm1	Y	140	1.00	38.91	5.6	5.9	0.3	EIREAFRVFI	_VFDKDGNGY(ph	3	612.2839	-0.3699	6.16E-07	143.3	119.5
F8WFW8	F8WFW8_RAT	Mitogen-a	Mapk1	Y	185	1.00	58.41	6.8	7.1	0.3	RVADPDHD	_VADPDHDHTGFL	3	768.6505	0.0469	1.63E-27	229.7	168.8
D3ZWN0	D3ZWN0_RAT	Protein Ple	Plekha6	T	401	0.96	13.53	5.4	5.7	0.3	ALEDKRHSF	_NGGGPT(ph)YQL	2	783.8405	-0.1471	2.96E-12	169.9	169.9
F1M2K6	F1M2K6_RAT	Uncharacterized protein (Fragi		Y	328	1.00	48.60	6.0	6.2	0.3	TVHDMDQI	_TVHDMDQFGQC	3	784.9912	-0.5876	2.02E-09	173.3	173.3
B5DF08	B5DF08_RAT	Protein So	Socs4, Socs4_predi	S	122	1.00	62.94	6.0	6.3	0.3	ARHSPGLPS	_HS(ph)PGLPS(ph	3	860.3692	0.2076	6.67E-02	62.9	62.9
B5DF08	B5DF08_RAT	Protein So	Socs4, Socs4_predi	T	118	1.00	62.94	6.0	6.3	0.3	RNCGARHSI	_HS(ph)PGLPS(ph	3	860.3692	0.2076	6.67E-02	62.9	62.9
Q2THW7	ZDHC5_RAT	Palmitoylt	Zdhhc5	S	529	1.00	42.17	5.8	6.0	0.3	RHPRLLPTG	_LLPTGPPHREPS(	3	854.4069	0.4085	1.62E-02	74.8	74.8
Q2THW7	ZDHC5_RAT	Palmitoylt	Zdhhc5	Y	533	1.00	25.76	5.8	6.0	0.3	LLPTGPPHR	_LLPTGPPHREPS(	3	854.4069	0.4085	1.62E-02	74.8	74.8
F1LM93	YES_RAT	Tyrosine-p	Yes1	Y	220	1.00	27.40	5.7	5.9	0.3	DNVKHYKIR	_KLDNGGY(ph)YI	2	740.8452	0.0334	9.89E-28	227.6	184.2
F1M4A0	F1M4A0_RAT	Protein Tjp	Tjp1	Y	1177	1.00	89.14	5.4	5.6	0.3	SGYEVHNRY	_YRPEAQPY(ph)A	2	812.8796	0.3755	3.31E-08	139.3	139.3
P21708	MK03_RAT	Mitogen-a	Mapk3, Erk1, Prkm	Y	205	1.00	59.66	7.1	7.3	0.3	RIADPEHDH	_IADPEHDHTGFLT	3	777.9943	0.1835	2.70E-27	222.4	150.0
D4A5V8	D4A5V8_RAT	Mitogen-a	Mapk9, rCG_34039	Y	185	1.00	49.05	6.2	6.4	0.3	FGLARTACT	_TACTNFMMTPY(	2	936.3981	-0.0964	2.24E-51	264.9	249.6
E7CXR8	E7CXR8_RAT	Epidermal	Egfr	Y	1091	0.88	8.60	5.0	5.3	0.3	LTEDNIDDT	_YSSDPTSVLTEDN	3	1188.5500	0.1441	5.16E-07	100.9	100.9
P11980	KPYM_RAT	Pyruvate k	Pkm, Pkm2, Pykm	Y	105	1.00	25.47	5.9	6.1	0.2	NVRAATESF	_AATESFASDPILY(	3	839.0875	0.4496	1.55E-04	119.8	119.8
D3ZZ55	D3ZZ55_RAT	Protein Ctr	Ctnnd1	Y	257	1.00	75.82	5.4	5.6	0.2	RPSMEGYR	_QDVY(ph)GPQP(	2	683.8112	-0.1209	3.91E-03	90.9	75.8
F1M8H9	F1M8H9_RAT	Mitogen-a	Mapk10	Y	223	1.00	29.93	6.0	6.3	0.2	FGLARTAGT	_TAGTSFMMTPY(	2	871.3881	0.1815	2.45E-15	176.9	176.9
D3ZJ50	D3ZJ50_RAT	Plakophilir	Pkp3, Pkp3_predict	Y	415	1.00	70.98	5.8	6.1	0.2	QEVQRHAT	_NLIY(ph)DNVDN	2	644.2845	0.5476	4.93E-12	179.6	71.0
E9PT59	E9PT59_RAT	Protein Arl	Arhgef5	Y	630	0.77	5.31	5.0	5.2	0.2	FSHLELPQR	_TAIY(ph)GSLIPR_	2	585.7996	-0.8227	4.87E-03	83.9	83.9
D3ZZ55	D3ZZ55_RAT	Protein Ctr	Ctnnd1	Y	96	1.00	26.46	6.5	6.7	0.2	SDLKLNGPC	_LNGPQDHNHLLY	3	685.6651	-0.3918	8.80E-09	155.6	78.2
D3ZMK9	D3ZMK9_RAT	Protein Pra	Pragmin, RGD1311	Y	391	1.00	42.46	6.1	6.3	0.2	QPPALPREA	_EAVQPEPIY(ph)A	2	806.3687	0.1204	1.49E-14	175.3	148.4
Q4QQV2	DOK1_RAT	Docking pr	Dok1	Y	361	0.97	15.15	6.6	6.8	0.2	QLLKTKLIDS	_LIDSKEDPIY(ph)D	3	840.0753	0.1948	1.26E-03	106.6	88.3
F1M2K6	F1M2K6_RAT	Uncharacterized protein (Fragi		Y	371	1.00	40.85	6.2	6.4	0.2	DLRSAVSPD	_SAVSPDLHITPIY(	3	612.2960	0.1958	2.32E-10	158.9	77.9
Q9WVT0	GP116_RAT	Probable G	Gpr116, Gprhep	S	1291	0.99	23.65	5.9	6.1	0.2	VQEALLHKF	_FSLSRWSS(ph)Q	2	805.3316	0.9295	7.34E-03	94.0	94.0
Q9WVT0	GP116_RAT	Probable G	Gpr116, Gprhep	S	1294	1.00	75.93	5.9	6.1	0.2	ALLHKFSLSF	_FSLSRWSS(ph)Q	2	805.3316	0.9295	7.34E-03	94.0	94.0
B2RZ33	B2RZ33_RAT	Nck1 prote	Nck1, Nck1_predict	Y	105	1.00	43.60	5.8	6.0	0.2	ASPADDSFV	_LY(ph)DLNMPAF	2	695.8275	0.8936	7.99E-06	89.3	43.6
P62630	EF1A1_RAT	Elongation	Eef1a1, Eef1a	Y	29	1.00	92.52	5.6	5.8	0.2	GHVDSGKST	_STTTGHLIY(ph)K	2	600.7867	-0.3391	1.37E-05	144.8	144.8
D4A563	D4A563_RAT	Protein RG	RGD1312026, RGD1	Y	631	1.00	60.03	5.8	6.0	0.2	SKNAIKVPIV	_NAIKVPIVINPNA	3	835.0848	-0.1426	3.06E-04	127.6	60.0
D4ADJ2	D4ADJ2_RAT	Activated (	Tnk2	Y	533	0.85	7.66	5.4	5.6	0.2	PPRPPQPAI	_KPTY(ph)DPVSEI	3	791.6931	-0.0892	7.14E-05	132.5	132.5
A2VD09	A2VD09_RAT	Abi1 prote	Abi1	Y	213	0.93	12.16	6.0	6.1	0.2	YKTLEPVKPI	_TLEPVKPPTVPNI	3	765.0360	-0.2186	5.94E-03	89.8	68.4
F1MAL5	F1MAL5_RAT	Protein Irs	Irs2	Y	817	1.00	47.85	6.2	6.3	0.2	SYKAPCTCG	_SYKAPCTCGGGD	3	933.7239	-0.4378	5.83E-06	124.7	120.4
F1M1Z5	F1M1Z5_RAT	Paxillin (Fr	Pxn	Y	88	0.98	19.91	6.4	6.6	0.2	GSRYAHQQ	_YAHQQPPS(ph)P	3	712.3009	0.1615	2.97E-13	182.6	113.6
F1MAL5	F1MAL5_RAT	Protein Irs	Irs2	Y	735	1.00	23.97	5.7	5.9	0.2	YKASSPAES	_ASSPAESSPEDSG	2	875.8349	0.3788	2.65E-22	216.3	169.4
Q9Z1T4	CNKR2_RAT	Connector	Cnksr2	Y	482	1.00	49.00	5.1	5.3	0.1	SLLRYMSNE	_IAQEEY(ph)MFQ	2	697.7942	0.6455	3.13E-02	55.3	49.0
O35889	AFAD_RAT	Afadin (Prc	Mllt4, Af6	Y	94	1.00	35.32	5.6	5.7	0.1	FRPDMRMI	_YSLY(ph)EVHVSC	2	759.8349	0.1269	2.25E-05	122.3	122.3
P85972	VINC_RAT	Vinculin (N	Vcl	Y	822	1.00	41.53	5.6	5.8	0.1	NISDPGLQK	_SFLDSGY(ph)R_	2	512.7104	-0.0653	2.00E-04	135.8	135.8
Q4QQV2	DOK1_RAT	Docking pr	Dok1	Y	408	0.96	13.36	6.4	6.6	0.1	KEEGYELPYI	_LKEEGYELPYNPA	3	905.4177	0.5238	8.53E-05	125.8	125.8
Q9Z272	GIT1_RAT	ARF GTPas	Git1	S	555	0.83	7.16	6.1	6.2	0.1	LQPFHSTELI	_LQPFHS(ph)TELE	3	1006.4542	0.3509	4.44E-06	130.1	44.4
D3ZDI5	D3ZDI5_RAT	Protein Arl	Arhgap12	S	238	1.00	77.99	6.1	6.2	0.1	EQMRATTP	_AT(ph)TPPNQGR	3	879.3780	-0.1098	2.11E-03	116.7	116.7
D3ZDI5	D3ZDI5_RAT	Protein Arl	Arhgap12	Y	241	1.00	95.63	6.1	6.2	0.1	RATTPPNQC	_AT(ph)TPPNQGR	3	879.3780	-0.1098	2.11E-03	116.7	116.7
D3ZWX4	D3ZWX4_RAT	Protein Nc	Nckipsd	Y	161	1.00	39.20	6.2	6.3	0.1	HSLPSSEHLC	_QHSLPSSEHLGTD	3	898.7606	0.0793	1.69E-03	70.1	70.1
D3ZE44	D3ZE44_RAT	Protein Sni	Snip	Y	464	1.00	84.49	6.4	6.5	0.1	AGKAGGMV	_GEGLY(ph)ADPY	2	913.9091	0.2944	2.69E-11	173.7	154.7
P19945	RLA0_RAT	60S acidic	Rplp0, Arbp	Y	24	1.00	91.85	5.6	5.7	0.1	WKSNYFLKI	_IIQLLDDY(ph)PK_	2	649.3256	-0.4367	2.03E-06	136.0	91.9
D3ZWN0	D3ZWN0_RAT	Protein Ple	Plekha6	Y	490	1.00	85.44	7.2	7.3	0.1	PSARFDRLP	_SEDIY(ph)ADPAA	2	840.8524	0.1890	2.78E-26	217.9	206.3
F1M4A0	F1M4A0_RAT	Protein Tjp	Tjp1	Y	1164	1.00	46.83	5.2	5.3	0.1	PRTSTLRHEI	_HEEQPTSGY(ph)	3	588.2442	-0.6634	1.81E-34	240.1	240.1
G3V6X4	G3V6X4_RAT	E3 ubiquiti	Nedd4, Nedd4a, rC	Y	365	0.97	15.83	5.3	5.4	0.1	VQVRLAEEL	_LTMY(ph)GNPAT	3	772.3423	-0.2910	1.14E-03	96.1	96.1
D3ZB46	D3ZB46_RAT	Hepatocyt	Foxa1	S	174	0.97	18.22	4.3	4.4	0.1	AKTFKRSYPI	_PPY(ph)S(ph)YISI	2	1134.5248	0.0361	7.74E-02	33.3	33.3
Q03114	CDK5_RAT	Cyclin-dep	Cdk5, Cdkn5	Y	15	0.98	16.89	5.9	5.9	0.1	_MQKYEKLI	_IGEGTY(ph)GTVI	2	626.2865	-0.2566	1.74E-11	161.7	157.4
D3ZH39	D3ZH39_RAT	Eph recept	Ephb3, Ephb3_prec	Y	787	0.93	14.17	6.0	6.1	0.1	FGLSRFLEDI	_FLEDDPSDPTY(p	2	1004.9248	0.4457	2.31E-37	247.0	247.0
D4ADU3	D4ADU3_RAT	Uncharact	Tenc1	Y	483	1.00	61.34	5.8	5.9	0.1	DGSLAHTRC	_GPLDGSPY(ph)A	2	734.3350	-0.0861	1.26E-18	194.4	194.4
F1LQM5	F1LQM5_RAT	Receptor-t	Ptpra	Y	827	1.00	54.80	6.4	6.5	0.1	FCYKVVQEY	_VVQEYIDAFSDY(	3	663.6274	-0.1694	2.47E-79	309.2	153.2
D3ZE44	D3ZE44_RAT	Protein Sni	Snip	Y	332	1.00	56.59	6.2	6.2	0.1	IQDRSIIKIYI	_KEPLY(ph)AAFPC	3	722.6842	0.0605	1.64E-05	139.1	108.2
F1LM93	YES_RAT	Tyrosine-p	Yes1	Y	424	1.00	24.93	6.5	6.6	0.1	KIADFGLAR	_LIEDNEY(ph)TAR	2	652.2819	-0.2387	1.71E-24	235.0	208.6
D3ZZ55	D3ZZ55_RAT	Protein Ctr	Ctnnd1	Y	904	1.00	32.67	6.4	6.4	0.0	PMSNMGSI	_SLDNNY(ph)STLI	2	753.3170	-0.0313	1.59E-36	249.8	195.0
F1M7V1	F1M7V1_RAT	Kin of IRRE	Kirrel	Y	756	1.00	89.62	5.9	5.9	0.0	PQAPPSGLE	_TPYEAY(ph)DPIG	2	667.2892	-0.0457	1.11E-08	152.7	152.7
B3Y9H3	B3Y9H3_RAT	S100 calciu	S100a10, p11, rCG_	Y	25	1.00	55.08	5.4	5.4	0.0	ETMMLTFH	_FAGEKNY(ph)LTI	2	625.7945	0.2081	1.94E-08	169.9	155.1
B1WBZ1	B1WBZ1_RAT	Embryonal	Efs, Efs_predicted,	Y	149	1.00	64.67	5.7	5.8	0.0	VNGIQLTAP	_DVGEVY(ph)DVP	2	833.3978	-0.3596	1.26E-02	64.7	64.7
F1M1Z5	F1M1Z5_RAT	Paxillin (Fr	Pxn	Y	181	0.99	19.39	5.8	5.9	0.0	EAESSPPLP(	_SPSGFSADEAESS	3	1207.9047	-0.0437	5.64E-16	138.4	118.2
D3ZJ50	D3ZJ50_RAT	Plakophilir	Pkp3, Pkp3_predict	Y	201	1.00	41.10	5.6	5.7	0.0	PVSFHERGC	_ADY(ph)DTLSLR_	2	567.2474	-0.0976	4.09E-07	163.4	163.4
F1LNP7	F1LNP7_RAT	Serine/thr	Prpf4b	Y	849	1.00	39.49	8.1	8.1	0.0	DFGSASHVA	_LCDFGSASHVAD	3	839.7087	-0.2125	2.22E-27	223.5	213.7
G3V7X2	G3V7X2_RAT	Secretogra	Scg2, rCG_23898	Y	459	0.99	19.42	5.4	5.4	0.0	MENVANQI	_SPYFPNQY(ph)SI	2	669.7794	-0.2673	1.14E-04	117.7	117.7
Q9Z340	PARD3_RAT	Partitionin	Pard3, Par3	Y	1127	1.00	75.41	6.8	6.8	0.0	ARPQSPREC	_EGHLMDTLY(ph)	3	528.9091	0.0209	4.30E-63	293.3	121.0
G3V760	G3V760_RAT	Intestinal	Ick	T	157	1.00	35.44	5.3	5.3	0.0	ADFGLAREII	_SRPPYT(ph)DY(p	2	801.3235	0.2678	2.42E-04	110.6	103.6
G3V760	G3V760_RAT	Intestinal	Ick	Y	159	1.00	37.26	5.3	5.3	0.0	FGLAREIRSF	_SRPPYT(ph)DY(p	2	801.3235	0.6142	2.42E-04	110.6	110.6
P41499	PTN11_RAT	Tyrosine-p	Ptpn11	Y	62	0.87	8.17	6.5	6.5	0.0	RNGAVTHIK	_IQNTGDY(ph)YD	2	908.3773	-0.0379	5.09E-80	319.8	319.8
D4A1Q5	D4A1Q5_RAT	Homeodor	Hipk1	Y	352	1.00	25.43	6.7	6.7	0.0	DFGSASHVS	_AVCSTY(ph)LQSI	2	632.7732	0.3615	3.96E-17	198.6	186.0
Q6AYT8	MPZL1_RAT	Myelin prc	Mpzl1	S	248	0.96	14.48	5.8	5.8	0.0	AGSHQGPV	_SPPSAGSHQGPV	3	930.0911	0.0336	1.34E-05	96.1	96.1
P39951	CDK1_RAT	Cyclin-dep	Cdk1, Cdc2, Cdc2a,	T	14	1.00	96.19	7.8	7.8	0.0	__MEDYIKII	_IGEGT(ph)Y(ph)C	2	673.2775	-0.1492	1.79E-41	257.0	121.0
P39951	CDK1_RAT	Cyclin-dep	Cdk1, Cdc2, Cdc2a,	Y	15	1.00	80.83	7.9	7.9	0.0	_MEDYIKIEI	_IGEGT(ph)Y(ph)C	2	673.2775	0.0848	9.53E-18	187.1	134.2
Q9Z1T4	CNKR2_RAT	Connector	Cnksr2	Y	452	1.00	126.48	5.8	5.8	0.0	LRPISMPVE	_LRPISMPVEYNW	3	868.0610	-0.1029	1.38E-12	185.1	182.2
G3V7P7	G3V7P7_RAT	Retinoblas	Rbl2, rCG_44271	S	1076	0.99	24.46	4.3	4.3	0.0	AMLSPREKI	_IFYYFSNS(ph)PS(	2	756.7960	0.4821	8.40E-02	37.0	37.0
G3V7P7	G3V7P7_RAT	Retinoblas	Rbl2, rCG_44271	S	1078	0.99	23.89	4.3	4.3	0.0	LSPREKIFYY	_IFYYFSNS(ph)PS(	2	756.7960	0.4821	8.40E-02	37.0	37.0
E7CXR8	E7CXR8_RAT	Epidermal	Egfr	S	1103	0.76	4.93	4.3	4.3	0.0	VPEYINQSV	_RPAGS(ph)VQNI	3	824.7397	-0.4823	1.78E-02	84.6	84.6
F1M1Z5	F1M1Z5_RAT	Paxillin (Fr	Pxn	S	119	0.81	6.23	4.3	4.3	0.0	VGSLCSRAG	_AGEEEHVYS(ph)I	2	793.8298	0.1198	1.51E-11	167.7	167.7
D3ZL68	D3ZL68_RAT	Protein Zc	Zcchc6	T	1290	1.00	77.28	4.3	4.3	0.0	FDLNHNLG	_KMT(ph)NFIM(o	2	554.7502	0.8799	5.52E-02	77.3	77.3
Q5G5J2	Q5G5J2_RAT	Putative u	LOC498971	T	15	1.00	59.57	4.3	4.3	0.0	_MNQAMH	_KLT(ph)S(ph)VPE	3	473.5626	-0.0723	6.43E-02	62.1	59.6
Q5G5J2	Q5G5J2_RAT	Putative u	LOC498971	S	16	1.00	59.57	4.3	4.3	0.0	MNQAMHP	_KLT(ph)S(ph)VPE	3	473.5626	-0.0723	6.43E-02	62.1	59.6
D4A4C0	D4A4C0_RAT	Uncharacterized protein		S	1643	0.97	11.52	4.3	4.3	0.0	KYIRLQVMS	_LQVMS(ph)LSPA	2	935.4817	0.1607	3.43E-02	58.3	58.3
D4A4E0	D4A4E0_RAT	Sortilin	Sort1	S	190	1.00	31.33	4.3	4.3	0.0	ENSGKVILT	_VILTAEVSGGS(pl	2	634.8159	-0.3324	4.37E-02	80.3	80.3
F1LM93	YES_RAT	Tyrosine-p	Yes1	Y	192	1.00	28.15	4.3	4.3	0.0	RGIFLVRESE	_GAY(ph)SLSIR_	2	473.7233	0.0423	2.76E-02	69.6	69.6
Q9JJ50	HGS_RAT	Hepatocyt	Hgs, Hrs, Hrs2	Y	216	1.00	70.81	4.3	4.3	0.0	PKFGIEKEVI	_VCEPCY(ph)EQLI	2	760.3015	0.2533	1.07E-02	70.8	70.8
D3ZHA0	D3ZHA0_RAT	Protein Fln	Flnc	S	2234	1.00	41.47	4.3	4.3	0.0	FPAVFGDFL	_LGS(ph)FGSITR_	2	509.2419	0.0023	1.67E-02	103.9	103.9
D4A0Y2	D4A0Y2_RAT	Protein RG	Dopey1, RGD13055	T	2372	1.00	37.37	4.3	4.3	0.0	RKRAKKNPE	_T(ph)LGWEPGHL	2	1127.4656	4.3498	7.54E-02	37.4	37.4
D4A0Y2	D4A0Y2_RAT	Protein RG	Dopey1, RGD13055	T	2383	1.00	37.37	4.3	4.3	0.0	GSARTLGW	_T(ph)LGWEPGHL	2	1127.4656	4.3498	7.54E-02	37.4	37.4
D4A0Y2	D4A0Y2_RAT	Protein RG	Dopey1, RGD13055	T	2386	1.00	37.37	4.3	4.3	0.0	RTLGWEPG	_T(ph)LGWEPGHL	2	1127.4656	4.3498	7.54E-02	37.4	37.4
Q6PW52	Q6PW52_RAT	GABA-A ga	Gabrg2, rCG_33820	T	315	0.83	10.43	4.3	4.3	0.0	VPARTSLGI	_TSLGITTVLTM(ox	2	1102.5840	2.0395	1.14E-02	70.7	70.7
D4AAK0	D4AAK0_RAT	Protein Zn	Zfp295, Znf295	T	316	0.93	12.49	4.3	4.3	0.0	KRPRPPVLS	_PPVLSLHSSSET(p	2	974.4950	0.1874	8.33E-02	46.7	46.7

D3ZKN4	D3ZKN4_RAT	Protein Wl	Whamm, RGD1563	T 340	1.00	62.04	4.3	4.3		0.0	AMPRLENLI	_LM(ox)LARET(ph	2	893.4594	2.9464	7.43E-02	62.0	62.0
F1MAL8	F1MAL8_RAT	Uncharacterized protein		T 376	0.78	8.57	4.3	4.3		0.0	SSKGSVIKRI	_RNQQSVTT(ph)E	2	848.8862	-0.4731	8.96E-03	79.7	79.7
G3V8L3	G3V8L3_RAT	Lamin A, is	Lmna, rCG_62695	S 390	0.93	10.42	4.3	4.3		0.0	AYRKLLEGEI	_LRLS(ph)PS(ph)P	2	701.3180	-0.1632	7.93E-03	91.4	91.4
P09760	FER_RAT	Tyrosine-p	Fer, Fert2, Flk	Y 402	1.00	76.53	4.3	4.3		0.0	KVQENDGK	_VQENDGKEPPPV	3	789.0163	0.1549	7.97E-03	76.5	76.5
F1LUF1	F1LUF1_RAT	Coiled-coil	Ccdc67	T 45	1.00	73.26	4.3	4.3		0.0	SNKKLDWEI	_KM(ox)RALET(ph	2	550.7677	4.0209	6.64E-02	73.3	73.3
Q9Z340	PARD3_RAT	Partitionin	Pard3, Par3	S 485	0.82	6.47	4.3	4.3		0.0	GLGFSITSRI	_DVTIGGS(ph)API	2	700.3471	0.2566	3.12E-17	188.1	188.1
G3V679	G3V679_RAT	Transferrir	Tfrc, rCG_52708	S 500	1.00	26.02	4.3	4.3		0.0	LDKVVLGTS	_VSAS(ph)PLLYT(p	2	778.3476	-0.7225	1.40E-02	65.2	58.2
Q715S9	TXND3_RAT	Thioredoxi	Nme8, Sptrx2, Txnc	T 522	0.99	20.21	4.3	4.3		0.0	VLDVLSSGN	_NVLDVLSSGM(o:	3	673.3365	1.1879	3.33E-02	47.0	47.0
E9PTT0	E9PTT0_RAT	Protein Zd	Zdhhc17	Y 60	1.00	39.35	4.3	4.3		0.0	DYSTWDIVI	_ATQYGIY(ph)ER_	2	590.7554	0.2249	1.02E-02	79.2	79.2
P70605	KCNN3_RAT	Small cond	Kcnn3	S 623	1.00	44.64	4.3	4.3		0.0	AIHQLRGVK	_KLS(ph)DQANT(p	2	846.3863	-3.7716	3.67E-02	70.4	70.4
P70605	KCNN3_RAT	Small cond	Kcnn3	T 628	1.00	50.14	4.3	4.3		0.0	RGVKMEQR	_KLS(ph)DQANT(p	2	846.3863	-3.7716	3.67E-02	70.4	70.4
P63170	DYL1_RAT	Dynein ligl	Dynll1, Dncl1, Dnclc	Y 65	0.90	9.70	4.3	4.3		0.0	YNPTWHCI\	_NFGSY(ph)VTHE	2	681.7899	0.3207	8.20E-36	244.0	244.0
D3ZDV9	D3ZDV9_RAT	Protein Ne	Nek4	T 667	1.00	72.32	4.3	4.3		0.0	GKSHTNEM	_DLVQLM(ox)T(pl	2	967.9565	0.1445	2.27E-02	72.3	72.3
D3ZDV9	D3ZDV9_RAT	Protein Ne	Nek4	T 669	1.00	72.32	4.3	4.3		0.0	SHTNEMRD	_DLVQLM(ox)T(pl	2	967.9565	0.1445	2.27E-02	72.3	72.3
D3ZSM1	D3ZSM1_RAT	Protein Ep	Epb4.1l2, RGD1563	S 707	1.00	30.91	4.3	4.3		0.0	KGESVITEEI	_ELS(ph)PGSGPGI	2	697.8192	-4.6365	7.85E-02	65.2	65.2
Q6AYT5	CF211_RAT	UPF0364 protein C6orf211 hor		T 71	1.00	101.30	4.3	4.3		0.0	KKAISLLSKLI	_NELQT(ph)DKPII	2	901.9686	-5.0356	2.35E-02	101.3	101.3
E9PTV0	E9PTV0_RAT	Protein Gu	Guk1	Y 74	0.93	13.08	4.3	4.3		0.0	HTTRNPRPC	_NPRPGEEDGKDY	3	708.3125	0.1041	2.86E-03	91.6	72.6
G8JLR5	G8JLR5_RAT	Synaptoph	Syp	Y 81	1.00	58.05	4.3	4.3		0.0	NIEVEFEYPI	_LHQVY(ph)FDAP	2	822.3680	0.7910	1.79E-04	107.7	107.7
G3V913	G3V913_RAT	Heat shock	Hspb1, rCG_21815	S 87	0.91	10.93	4.3	4.3		0.0	LAAPAFSRA	_QLSS(ph)GVSEIR	2	578.2739	-0.1874	6.05E-02	78.7	78.7
F1LYT6	F1LYT6_RAT	Uncharacterized protein (Fragı		T 90	0.98	17.68	4.3	4.3		0.0	LEVSFTTVTI	_FLGT(ph)MISWD	2	639.2854	0.9228	1.40E-02	80.8	80.8
D3Z9K9	D3Z9K9_RAT	Glyceroph	Gdpd2, Gdpd2_pre	S 243	1.00	60.76	4.3	4.3		0.0	GHRGAPMI	_PGLVGHRGAPM	3	831.7230	-0.6798	8.25E-02	60.8	60.8
D3ZDQ1	D3ZDQ1_RAT	Protein Cy	Cyp2c79	S 422	1.00	31.43	4.3	4.3		0.0	FDPSHFLDE	_S(ph)DY(ph)FFPI	3	539.1581	5.6694	6.67E-02	31.4	31.4
D3ZDQ1	D3ZDQ1_RAT	Protein Cy	Cyp2c79	S 429	1.00	31.43	4.3	4.3		0.0	DENGKVKK:	_S(ph)DY(ph)FFPI	3	539.1581	5.6694	6.67E-02	31.4	31.4
D3ZGU4	D3ZGU4_RAT	Protein Ol	Olr332	S 19	1.00	69.86	4.3	4.3		0.0	GRNSTTVTF	_FILLGFS(ph)DQP	2	793.4049	1.2385	3.91E-02	69.9	69.9
D3ZRU4	D3ZRU4_RAT	Uncharacterized protein		S 219	1.00	39.09	4.3	4.3		0.0	DADIDLKGV	_ILLIS(ph)EHLKS(p	2	1053.5254	0.2031	4.11E-02	66.6	66.6
D3ZRU4	D3ZRU4_RAT	Uncharacterized protein		S 224	1.00	41.10	4.3	4.3		0.0	LKGVAKILLI:	_ILLIS(ph)EHLKS(p	2	1053.5254	0.2031	4.11E-02	66.6	66.6
D4A454	D4A454_RAT	Protein RG	RGD1306091	S 728	0.80	6.10	4.3	4.3		0.0	LDVRKLHG/	_LHGAQVPTKPS(p	2	720.3740	0.9241	7.58E-03	86.1	86.1
D4AAU7	D4AAU7_RAT	Protein Lrr	Lrrc9	S 535	0.94	13.87	4.3	4.3		0.0	FLQQKYKEE	_SPSES(ph)DLY(pl	2	607.2123	-4.5438	7.95E-02	30.0	30.0
E9PU24	E9PU24_RAT	Uncharacterized protein		S 721	1.00	65.56	4.3	4.3		0.0	VAVLREVKY	_KS(ph)NIPDS(ph)	3	559.9265	-5.4550	2.74E-02	65.6	65.6
E9PU24	E9PU24_RAT	Uncharacterized protein		S 726	1.00	65.56	4.3	4.3		0.0	EVKYLLMLK	_KS(ph)NIPDS(ph)	3	559.9265	-5.4550	2.74E-02	65.6	65.6
F1M8U2	F1M8U2_RAT	Protein Ab	Ablim3	S 138	0.87	7.29	4.3	4.3		0.0	SGKECVCQT	_ECVCQTCSQSM(	3	683.5351	-0.1753	6.59E-02	32.2	32.2
F1M8U2	F1M8U2_RAT	Protein Ab	Ablim3	S 139	0.99	21.13	4.3	4.3		0.0	GKECVCQT(	_ECVCQTCSQSM(	3	683.5351	-0.1753	6.59E-02	32.2	32.2
Q9JHB5	TSNAX_RAT	Translin-as	Tsnax, Trax	S 30	1.00	27.39	4.3	4.3		0.0	HDNFPHNC	_DAS(ph)SS(ph)S(	2	790.2813	0.6311	8.02E-02	38.2	38.2
Q9JHB5	TSNAX_RAT	Translin-as	Tsnax, Trax	S 32	0.85	6.89	4.3	4.3		0.0	NFPHNQRR	_DAS(ph)SS(ph)S(	2	790.2813	0.6311	8.02E-02	38.2	38.2
Q9JHB5	TSNAX_RAT	Translin-as	Tsnax, Trax	S 33	0.85	6.89	4.3	4.3		0.0	FPHNQRRE(	_DAS(ph)SS(ph)S(	2	790.2813	0.6311	8.02E-02	38.2	38.2
Q9QZX1	Q9QZX1_RAT	Nucleolin-ı	Norp, NRP	S 433	1.00	69.55	4.3	4.3		0.0	FEDAVEIRL\	_DGKS(ph)KGIAYI	2	768.3789	1.0251	3.66E-02	90.6	90.6
Q9R141	S22A4_RAT	Solute carr	Slc22a4, Octn1	S 323	1.00	34.65	4.3	4.3		0.0	APAVIFDPLI	_M(ox)NGIM(ox)/	3	885.7652	1.1451	7.30E-02	34.6	34.6
D3Z8Z9	D3Z8Z9_RAT	Protein Ck	Ckap2l	T 479	1.00	33.23	4.3	4.3		0.0	DRRKQLEEV	_T(ph)Y(ph)KRPPI	2	720.8292	-3.8768	8.56E-02	33.2	33.2
D3Z9K9	D3Z9K9_RAT	Glyceroph	Gdpd2, Gdpd2_pre	T 240	1.00	60.76	4.3	4.3		0.0	GLVGHRGA	_PGLVGHRGAPM	3	831.7230	-0.6798	8.25E-02	60.8	60.8
D3ZDQ1	D3ZDQ1_RAT	Protein Cy	Cyp2c79	T 430	1.00	31.43	4.3	4.3		0.0	ENGKVKKSI	_S(ph)DY(ph)FFPI	3	539.1581	5.6694	6.67E-02	31.4	31.4
F1LN42	F1LN42_RAT	Protein Tn	Tns1	T 1416	0.89	9.26	4.3	4.3		0.0	RMSVGDRA	_AGSLPNYAT(ph)I	2	693.3267	-0.2000	3.50E-02	57.2	57.2
Q66HC3	CI072_RAT	Protein C9orf72 homolog		T 337	1.00	29.09	4.3	4.3		0.0	HEHIYNQRF	_SELT(ph)AFWR_	2	545.2419	-0.9905	6.65E-02	65.0	65.0
D3ZUP8	D3ZUP8_RAT	Protein Lm	Lmbrd2, RGD15639	Y 290	1.00	39.02	4.3	4.3		0.0	CPTDYQEKI	_NMDDY(ph)EDFI	2	828.7978	-0.1294	2.10E-02	39.0	39.0
F1LXK8	F1LXK8_RAT	Protein LO	LOC100362634	Y 1513	1.00	51.13	4.3	4.3		0.0	PPATPSSMI	_LLY(ph)IFPGNLLE	3	565.9672	2.5344	2.45E-02	51.1	51.1
P38983	RSSA_RAT	40S ribosо	Rpsa, Lamr1	Y 139	0.75	5.06	4.3	4.3		0.0	VTDPRADH(	_ADHQPLTEASY(p	3	1026.1530	-0.2120	1.84E-12	153.6	153.6
Q9Z272	GIT1_RAT	ARF GTPas	Git1	Y 554	0.96	13.94	6.1	6.1		0.0	RLQPFHSTE	_LQPFHSTELEDD/	3	979.7988	0.5376	3.31E-08	149.4	149.4
P19139	CSK21_RAT	Casein kin	Csnk2a1	T 314	0.99	18.64	7.1	7.1		0.0	DFLDKLLRYI	_LT(ph)AREAMEH	4	692.0453	-0.6752	6.61E-02	30.4	30.4
D4A2U1	D4A2U1_RAT	Homeodor	Hipk3	T 358	0.92	10.81	6.6	6.6		0.0	IDFGSASHV	_TVCST(ph)YLQSF	2	647.7785	-0.0643	2.34E-08	163.5	163.5
P81128	RHG35_RAT	Rho GTPas	Arhgap35, Grlf1	Y 1105	1.00	47.34	7.1	7.1		0.0	PMDAVVKP	_NEEENIY(ph)SVP	3	676.2845	0.0704	1.26E-62	297.5	218.6
F1M2K6	F1M2K6_RAT	Uncharacterized protein (Fragı		T 433	0.91	10.03	6.1	6.1		0.0	TLTYQRNN\	_NNYALNTAAT(pl	2	1278.0894	0.2814	6.22E-05	142.4	139.5
D3ZE30	D3ZE30_RAT	Breast can	Bcar1	Y 347	1.00	27.59	5.8	5.8		0.0	YDTPRHLLA	_HLLAPGSQDIY(p	3	652.9908	-0.0291	1.29E-03	85.9	85.9
Q63470	DYR1A_RAT	Dual speci	Dyrk1a, Dyrk	Y 321	1.00	78.84	7.4	7.4		0.0	VDFGSSCQI	_IYQY(ph)IQSR_	2	575.7683	0.0328	6.62E-16	215.4	177.4
D3ZJC8	D3ZJC8_RAT	Cyclin-dep	Cdk2	Y 15	1.00	80.83	7.9	7.9		0.0	_MENFQKV	_IGEGT(ph)Y(ph)(	2	673.2775	0.0848	1.91E-15	187.1	134.2
F1LMJ2	F1LMJ2_RAT	Mitogen-a	Mapk7	T 219	0.84	7.17	6.2	6.2		0.0	ARGLCTSPA	_GLCTSPAEHQYFI	3	814.3482	-0.1076	2.68E-05	137.4	137.4
D3ZJC8	D3ZJC8_RAT	Cyclin-dep	Cdk2	T 14	1.00	96.19	7.8	7.8		0.0	__MENFQK	_IGEGT(ph)Y(ph)(	2	673.2775	-0.1492	2.99E-51	270.8	121.0
F1LZ81	F1LZ81_RAT	Uncharacterized protein (Fragı		Y 1906	0.80	7.01	5.3	5.3		0.0	EPVRKESKT	_TPPPYSVY(ph)ER	2	644.7841	0.3099	1.89E-02	64.4	51.4
F1M2K6	F1M2K6_RAT	Uncharacterized protein (Fragı		Y 1125	1.00	41.93	6.3	6.3		0.0	STDYSTQYG	_STTNY(ph)VDFY:	2	753.3134	0.1436	4.22E-12	168.8	168.8
F1M1Z5	F1M1Z5_RAT	Paxillin (Fr	Pxn	Y 118	1.00	37.54	6.7	6.7		0.0	SVGSLCSRA	_AGEEEHVY(ph)SI	3	614.9401	-0.0632	7.74E-41	244.2	201.0
F1MAL5	F1MAL5_RAT	Protein Irs	Irs2	Y 672	1.00	27.01	6.6	6.5		0.0	AALRSGGPI	_SDDY(ph)MPMS	2	896.8621	-0.0649	1.00E-21	208.5	192.7
Q9Z340	PARD3_RAT	Partitionin	Pard3, Par3	Y 1080	1.00	125.72	6.6	6.6		0.0	AKTREFRER	_ERDY(ph)AEIQDI	3	553.5701	-0.0612	3.46E-11	160.7	125.7
Q9Z340	PARD3_RAT	Partitionin	Pard3, Par3	Y 1242	1.00	76.11	6.2	6.2		0.0	RKNASSVSC	_KNASSVSQDSWE	3	932.4030	-0.1410	3.30E-10	157.6	157.6
F1MAL5	F1MAL5_RAT	Protein Irs	Irs2	Y 539	0.79	6.58	5.6	5.5		-0.1	ETPPARDGS	_DGSGGELYGY(pl	3	842.3396	0.7173	3.24E-03	91.5	91.5
F1M6V1	F1M6V1_RAT	Heterochr	Hp1bp3	Y 377	0.82	9.50	5.8	5.8		-0.1	LKKYVLENH	_YVLENHPGTNSN	3	780.0277	0.4300	3.39E-03	78.3	65.5
D3ZUD6	D3ZUD6_RAT	Focal adhe	Ptk2	Y 895	0.83	6.83	6.2	6.1		-0.1	REDGSFQGI	_GSIDREDGSFQGI	4	885.9191	-0.2242	8.86E-06	93.3	78.8
F1M1Z5	F1M1Z5_RAT	Paxillin (Fr	Pxn	S 83	1.00	37.12	6.2	6.1		-0.1	QWQPSGSF	_YAHQQPPS(ph)P	3	712.3009	0.1441	9.84E-09	162.8	162.8
D4A2U1	D4A2U1_RAT	Homeodor	Hipk3	Y 359	0.98	16.67	6.5	6.5		-0.1	DFGSASHVS	_TVCSTY(ph)LQSF	2	647.7785	0.2072	1.35E-12	192.5	152.7
P81128	RHG35_RAT	Rho GTPas	Arhgap35, Grlf1	S 1085	0.93	11.29	6.9	6.8		-0.1	SMSSSPWN	_SMSSSPWMPQD	3	1103.1309	-0.1253	5.98E-10	142.5	142.5
F1LMX3	F1LMX3_RAT	Ephrin typ	Epha8	Y 766	1.00	34.55	5.3	5.2		-0.1	DFGLSRALE	_ALEDDPEAAY(ph	2	844.8562	0.0894	5.13E-15	176.4	176.4
D3ZH39	D3ZH39_RAT	Eph recept	Ephb3, Ephb3_prec	Y 609	0.97	15.35	5.6	5.5		-0.1	QYVAPRMK	_VYIDPFTY(ph)ED	2	1004.4404	-0.3914	1.10E-10	167.7	167.7
F1MAL5	F1MAL5_RAT	Protein Irs	Irs2	Y 650	1.00	120.50	6.6	6.5		-0.1	GSHKSSSSN	_SSSSNLGADDGY(	2	1139.4794	0.0021	3.76E-76	309.8	309.8
B2RZ74	B2RZ74_RAT	Protein Sn	Snrnp70, Snrp70, Sı	Y 126	1.00	70.03	5.9	5.8		-0.1	NYDTTESKL	_EFEVY(ph)GPIKR	2	659.3156	0.4267	1.73E-02	70.0	70.0
F1LVS4	F1LVS4_RAT	Protein Ple	Plekha7	Y 583	1.00	41.42	5.5	5.4		-0.1	TVSAPSLHG	_SADDTY(ph)LQLI	2	681.3211	0.2949	2.51E-15	186.9	186.9
D3ZZ55	D3ZZ55_RAT	Protein Ctr	Ctnnd1	Y 228	1.00	29.54	6.7	6.6		-0.1	YGRHYEDG\	_HYEDGYPGGSDN	3	685.2690	0.0220	2.46E-62	297.5	186.8
F1LUB9	F1LUB9_RAT	Protein RG	RGD1564046	S 53	0.85	7.70	7.0	6.9		-0.1	ENEIAARKR	_M(ox)KTHS(ph)N	3	527.2115	0.8949	4.81E-02	64.7	64.7
Q07936	ANXA2_RAT	Annexin A	Anxa2, Anx2	Y 317	0.89	9.02	5.9	5.7		-0.1	KIRSEFKRKY	_SLYY(ph)FIQQDT	2	743.3367	-0.1968	3.56E-15	176.2	158.4
F1M2K6	F1M2K6_RAT	Uncharacterized protein (Fragı		Y 434	0.98	16.22	7.0	6.8		-0.1	LTYQRNNY/	_NNYALNTAATY(p	3	852.3953	-0.0103	1.86E-12	180.8	161.2
Q9WTQ6	TRIB3_RAT	Tribbles hc	Trib3, Nipk	T 4	1.00	38.05	6.3	6.1		-0.2	__________	_AT(ph)SLAAS(ph	2	739.7910	-0.4916	5.47E-02	59.7	59.7
Q9WTQ6	TRIB3_RAT	Tribbles hc	Trib3, Nipk	S 9	0.78	5.45	6.3	6.1		-0.2	_______MF	_AT(ph)SLAAS(ph	2	739.7910	-0.4916	5.47E-02	59.7	59.7
F1M2M0	F1M2M0_RAT	Protein Dy	Dyrk4	Y 380	1.00	30.32	6.2	6.0		-0.2	IDFGSSCYEI	_VYTY(ph)IQSR_	2	555.2550	-0.0337	1.37E-15	211.5	174.4
D3ZWN0	D3ZWN0_RAT	Protein Ple	Plekha6	Y 360	0.89	11.16	6.5	6.3		-0.2	VPEYYSPYS:	_RVPEYYSPYSSQY	4	1141.2409	-0.3440	2.87E-03	74.6	74.6
Q63538	MK12_RAT	Mitogen-a	Mapk12, Sapk3	T 183	0.91	10.26	5.7	5.5		-0.2	KILDFGLAR(	_QADSEMT(ph)G\	2	768.8236	-0.4341	1.68E-05	127.0	93.3
P81128	RHG35_RAT	Rho GTPas	Arhgap35, Grlf1	Y 1087	1.00	27.96	6.9	6.7		-0.2	SSSPWMPC	_SMSSSPWMPQD	3	1103.1309	-0.1974	2.80E-12	152.5	125.3
E1U7Q0	E1U7Q0_RAT	Cyclin-dep	Cdkl5	Y 171	1.00	33.49	5.9	5.7		-0.2	FARNLSEGN	_NLSEGNNANYTE	2	998.4258	-0.0452	1.01E-38	261.2	261.2
Q5U2U2	CRKL_RAT	Crk-like prı	Crkl	Y 251	1.00	47.60	5.0	4.7		-0.2	GPVFAKAIQ	_RVPCAY(ph)DK_	2	544.7334	0.0423	1.81E-02	88.8	47.6
D4A3Y6	D4A3Y6_RAT	Uncharacterized protein		T 82	0.93	13.59	4.5	4.3		-0.2	AKFQIPLPT(	_FQIPLPTGTALPH	2	864.9453	-0.7192	7.67E-02	49.5	49.5
F1LN42	F1LN42_RAT	Protein Tn	Tns1	Y 1414	1.00	34.24	5.6	5.4		-0.2	KRRMSVGD	_AGSLPNY(ph)ATI	2	693.3267	-0.3661	1.20E-08	150.5	128.1
D3ZUD6	D3ZUD6_RAT	Focal adhe	Ptk2	Y 610	1.00	31.00	6.6	6.4		-0.2	KLGDFGLSR	_YMEDSTY(ph)Y(p	2	823.2983	0.0648	2.86E-21	208.3	203.1
P34058	HS90B_RAT	Heat shock	Hsp90ab1, Hsp84, I	S 255	1.00	59.67	6.0	5.7		-0.3	EDKEDEEKP	_IEDVGS(ph)DEEI	3	649.2685	-0.0017	6.60E-03	113.8	113.8
F1LVS4	F1LVS4_RAT	Protein Ple	Plekha7	Y 397	1.00	34.40	6.3	6.0		-0.3	LPRQGPSQI	_QGPSQPPSFLEN'	3	713.6684	-0.0528	1.05E-05	139.3	139.3
G3V7X2	G3V7X2_RAT	Secretogra	Scg2, rCG_23898	S 558	0.86	7.85	5.7	5.3		-0.4	EQLEQAIKEI	_EHLGQGSS(ph)Q	2	770.3109	0.1577	6.62E-02	72.5	72.5
P18265	GSK3A_RAT	Glycogen s	Gsk3a	Y 279	0.97	15.60	8.0	7.6		-0.4	FGSAKQLVF	_GEPNVSY(ph)ICS	2	681.2814	0.1201	1.78E-15	186.8	86.8
D3ZGG9	D3ZGG9_RAT	Protein Xc	Xcr1	S 142	0.95	9.92	6.7	6.2		-0.6	FFLTIMTIHF	_YM(ox)SVVS(ph)	3	714.6392	-0.0503	3.00E-02	47.3	47.3
D3ZGG9	D3ZGG9_RAT	Protein Xc	Xcr1	S 145	0.80	6.80	6.7	6.2		-0.6	TIMTIHRYN	_YM(ox)SVVSPIS(	3	714.6392	-0.2122	3.00E-02	47.3	39.5
D3ZGG9	D3ZGG9_RAT	Protein Xc	Xcr1	S 146	0.80	6.80	6.7	6.2		-0.6	IMTIHRYMS	_YM(ox)SVVSPIS(	3	714.6392	-0.2122	3.00E-02	47.3	39.5
D3ZGG9	D3ZGG9_RAT	Protein Xc	Xcr1	T 151	0.96	14.20	6.7	6.2		-0.6	RYMSVVSPI	_YM(ox)SVVSPISS	3	714.6392	-0.2049	3.00E-02	47.3	41.0
D4A5P0	D4A5P0_RAT	Sodium/hy	Slc9a5	Y 317	1.00	66.27	5.0	4.3		-0.7	SAILAVTMC	_KY(ph)VEANIS(pl	2	674.7885	2.9184	2.16E-02	66.3	66.3
D4A5P0	D4A5P0_RAT	Sodium/hy	Slc9a5	S 323	1.00	66.27	5.0	4.3		-0.7	TMCGLGCK	_KY(ph)VEANIS(pl	2	674.7885	2.9184	2.16E-02	66.3	66.3
D4A924	D4A924_RAT	Protein No	Nop14, RGD13056C	S 732	1.00	75.57	5.3	4.3		-1.0	IVTPHQALL	_S(ph)LPQELQELS	2	690.3320	-1.3824	1.06E-02	88.1	88.1
G3V7X2	G3V7X2_RAT	Secretogra	Scg2, rCG_23898	Y 454	0.94	13.00	5.4	4.3		-1.1	LNVLGMEN	_SPY(ph)FPNQYSI	2	669.7794	0.1739	2.37E-02	54.2	39.8
G3V617	G3V617_RAT	Mitogen a	Mapk14, rCG_6107	Y 182	1.00	46.45	6.0	4.8		-1.1	LDFGLARHT	_HTDDEMTGY(ph	3	525.8764	0.5639	2.45E-04	120.8	111.6
Q9Z1T4	CNKR2_RAT	Connector	Cnksr2	S 510	0.80	8.56	5.6	4.3		-1.3	KSKKKGDKS	_S(ph)TSPTHYS(pl	3	759.6723	0.0481	6.55E-05	131.6	115.4
D3ZM60	D3ZM60_RAT	Protein Pp	Ppp1r3a, RGD1561	S 861	1.00	52.60	5.6	4.3		-1.3	LDLHLGVLP	_AILS(ph)ANADLE	3	672.6532	-1.8508	5.46E-02	52.6	52.6
D3ZM60	D3ZM60_RAT	Protein Pp	Ppp1r3a, RGD1561	S 873	1.00	52.60	5.6	4.3		-1.3	AILSANADLI	_AILS(ph)ANADLE	3	672.6532	-1.8508	5.46E-02	52.6	52.6
Q6IRS6	Q6IRS6_RAT	Fetub prot	Fetub	S 220	0.95	9.23	5.7	4.3		-1.4	LVKVTKATT	_ATT(ph)QWVVG	3	807.6601	0.4951	1.54E-02	33.0	33.0
Q6IRS6	Q6IRS6_RAT	Fetub prot	Fetub	Y 221	0.89	6.12	5.7	4.3		-1.4	VKVTKATTC	_ATT(ph)QWVVG	3	807.6601	0.4951	1.54E-02	33.0	33.0
Q6IRS6	Q6IRS6_RAT	Fetub prot	Fetub	Y 225	1.00	19.68	5.7	4.3		-1.4	KATTQWVV	_ATT(ph)QWVVG	3	807.6601	0.4951	1.54E-02	33.0	33.0
P18211	HB2D_RAT	Rano class	RT1-Db1	S 145	0.99	20.27	5.8	4.3		-1.5	PSKTQPLEH	_TQPLEHHNLLVC:	3	1068.4298	0.6311	7.47E-02	32.7	32.7
P18211	HB2D_RAT	Rano class	RT1-Db1	S 147	0.97	14.99	5.8	4.3		-1.5	KTQPLEHHI	_TQPLEHHNLLVC:	3	1068.4298	0.6311	7.47E-02	32.7	32.7
P18211	HB2D_RAT	Rano class	RT1-Db1	S 153	1.00	28.62	5.8	4.3		-1.5	HHNLLVCSV	_TQPLEHHNLLVC:	3	1068.4298	0.6311	7.47E-02	32.7	32.7
F2Z3Q9	F2Z3Q9_RAT	COP9 signa	Cops2	S 228	1.00	31.07	5.8	4.3		-1.5	TAQKNNKK	_ALY(ph)EQS(ph)I	2	681.2987	0.3561	6.68E-02	31.1	31.1
D4A5G8	D4A5G8_RAT	Protein LO	LOC685778, LOC10	S 327	1.00	64.64	5.9	4.3		-1.6	SKSDPIMLLI	_M(ox)VNS(ph)NI	2	805.3327	-0.3345	1.59E-02	64.6	64.6
D4A5G8	D4A5G8_RAT	Protein LO	LOC685778, LOC10	S 331	1.00	64.64	5.9	4.3		-1.6	PIMLLKDRN	_M(ox)VNS(ph)NI	2	805.3327	-0.3345	1.59E-02	64.6	64.6
G3V8S2	G3V8S2_RAT	SHC-transf	Shc1, rCG_62677	Y 423	0.99	18.86	6.3	4.3		-2.0	PPPPCPGRE	_ELFDDPSY(ph)VI	2	995.4457	-0.0920	1.11E-79	319.3	277.9
P81128	RHG35_RAT	Rho GTPas	Arhgap35, Grlf1	S 1106	0.77	5.95	7.0	4.3		-2.7	MDAVVKPR	_NEEENIYS(ph)VP	3	676.2845	-0.0532	1.23E-03	95.6	95.6
